# Glucose Levels Alter the Mga Virulence Regulon in the Group A Streptococcus

**DOI:** 10.1038/s41598-018-23366-7

**Published:** 2018-03-21

**Authors:** Kayla M. Valdes, Ganesh S. Sundar, Ashton T. Belew, Emrul Islam, Najib M. El-Sayed, Yoann Le Breton, Kevin S. McIver

**Affiliations:** 1 Department of Cell Biology & Molecular Genetics and Maryland Pathogen Research Institute, University of Maryland, College Park (UMCP), College Park, Maryland USA; 2 Center for Bioinformatics and Computation Biology, UMCP, College Park, MD USA

## Abstract

Many bacterial pathogens coordinately regulate genes encoding important metabolic pathways during disease progression, including the phosphoenolpyruvate (PEP)-phosphotransferase system (PTS) for uptake of carbohydrates. The Gram-positive Group A Streptococcus (GAS) is a pathogen that infects multiple tissues in the human host. The virulence regulator Mga in GAS can be phosphorylated by the PTS, affecting Mga activity based on carbohydrate availability. Here, we explored the effects of glucose availability on the Mga regulon. RNA-seq was used to identify transcriptomic differences between the Mga regulon grown to late log phase in the presence of glucose (THY) or after glucose has been expended (C media). Our results revealed a correlation between the genes activated in C media with those known to be repressed by CcpA, indicating that C media mimics a non-preferred sugar environment. Interestingly, we found very little overlap in the Mga regulon from GAS grown in THY versus C media beyond the core virulence genes. We also observed an alteration in the phosphorylation status of Mga, indicating that the observed media differences in the Mga regulon may be directly attributed to glucose levels. Thus, these results support an *in vivo* link between glucose availability and virulence regulation in GAS.

## Introduction

Coordinated expression of virulence factors is essential for bacterial pathogens to successfully colonize and elicit an infection in the host. Since expression of virulence genes is often linked to the availability of essential nutrients such as carbohydrates, pathogenic bacteria often utilize carbon catabolism regulatory pathways to sense the presence of preferred carbohydrates and repress the genes involved in alternative sugar utilization^1,2^. In fact, many pathogens have been shown to coordinately regulate the expression of carbohydrate metabolism genes with disease progression during *in vivo* studies^3–9^.

The phosphoenolpyruvate (PEP)-phosphotransferase system (PTS) allows bacteria to uptake carbohydrates and monitor carbon utilization^1^ via two cytoplasmic enzymes, EI (*ptsI*) and Hpr (*ptsH*), and several sugar-specific EII components. Each EII comprises two cytosolic subunits (EIIAB), an integral membrane transporter (EIIC), and sometimes a second membrane component (EIID). Phosphotransfer begins when the PEP generated from glycolysis transfers its phosphate to EI, which then is transferred to Hpr on His-15, then a a sugar-specific EIIA, then EIIB and finally to the transported sugar via its EIIC^1^. When Gram-positive bacteria are in nutrient rich conditions, Hpr is phosphorylated at Ser-46 by the kinase, HprK, allowing HprSer~P to dimerize with the carbon catabolite protein, CcpA, and elicit carbon catabolite repression (CCR) by binding to catabolite response elements (*cre*) found in promoter sequences^10^. In the absence of a preferred carbon source, Hpr-His15~P and EIIB~P are capable of phosphorylating histidines within PTS regulatory domains (PRDs) of transcriptional regulators (LicT, MtlR, LevR, *etc*.), regulating their activity and the expression of non-preferred sugar operons^1^. Thus, the PTS represents a signal transduction network through which Gram-positive pathogens alter gene regulation in response to carbohydrate utilization.

The Group A Streptococcus (GAS, *Streptococcus pyogenes*) is a Gram-positive pathogen that can colonize a variety of tissues in the human host, resulting in both life-threatening invasive as well as benign diseases. Each year around the world, GAS elicits over 700 million self-limiting infections and results in more than 500,000 deaths due to invasive infections and nonsuppurative sequelae^11^. Both *ex vivo* and *in vivo* studies have established that GAS exhibits significant changes in its transcriptome during infection^4,12–15^. As a fastidious fermentative organism that relies heavily on carbohydrates, metabolic genes under CCR are induced *in vivo* and are often required for full virulence in GAS^16–21^, directly linking GAS carbohydrate metabolism and virulence. Here we use the M1T1 strain 5448, a representative of one of the most prevalent serotypes of GAS isolated from invasive forms of infections worldwide.

GAS utilizes global transcriptional regulators such as the ubiquitous stand-alone regulator Mga to coordinate transcriptome changes impacting virulence^22^. Mga is critical for multiple *in vivo* phenotypes, including biofilm formation, growth in whole human blood and soft tissue, resistance to phagocytosis, and attachment to keratinocytes^4,12,23–26^. Mga regulates approximately 10% of the GAS genome during exponential phase of growth in rich medium (THY), including transcription of several sugar transport and utilization operons^27^. The ‘core’ Mga regulon consists of virulence genes critical for attachment and immune evasion such as M protein (*emm*), the *emm*-superfamily (*mrp*, *enn*, *arp*, *etc*.), C5a peptidase (*scpA*), and fibronectin-binding protein (*fba)*^28^. The Mga regulon was found to be expressed during the acute phase of GAS-mediated pharyngitis in macaques concurrently with the PTS and sugar metabolism operons^15^. Taken together, these data underscore that Mga and its virulence regulon are linked to carbohydrate utilization.

Mga belongs to a novel family of PTS regulatory domain (PRD)-containing virulence regulators (PCVR) that allows the PTS to directly phosphorylate and affect the activity of Mga based on carbohydrate availability^3,29^. Genetic, biochemical, and structural studies on the homologous *Bacillus anthracis* toxin regulator, AtxA, indicate that it is also a PCVR^30–33^. Two reiterative PRD domains (PRD-1 and PRD-2) of Mga allow PTS phosphorylation to impact its function likely through controlling dimerization of the carboxy-terminal EIIB^Gat^-like domain comparable to AtxA and other Gram-positive sugar-specific PRD-containing activators^3,24,31,32,34^. Although it is known that Mga activity is modulated by the PTS, we still do not know whether carbohydrate availability affects Mga-dependent gene regulation. In this study, we explored the effects of glucose availability on Mga and its impact on the Mga regulon. RNA-Seq was used to identify transcriptomic differences between the Mga regulon grown to late exponential phase either in the presence (THY) or absence (C media) of glucose. We observed that Mga was differentially phosphorylated in THY in comparison to C media, which led to a high degree of plasticity of the regulon that is correlated to glucose availability.

## Results

### Transcriptome of M1T1 5448 growing in presence or absence of glucose

**Figure 1:**
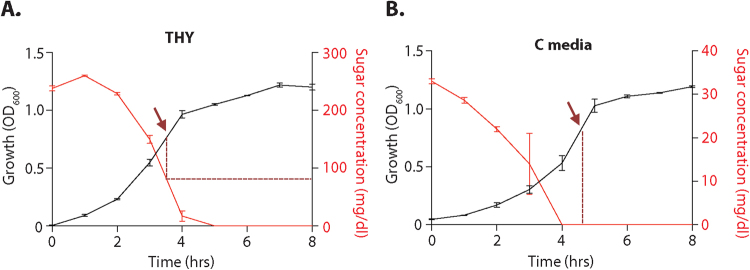
Comparison of glucose concentration over growth of GAS in THY and C media. M1T1 GAS 5448 was grown in either THY (**A**) or C media (**B**), with growth (black, OD_600_) and total sugar (glucose) concentration (red, mg/dl measured every hour as described in Methods). Data represent the average of three replicates, shown with the standard error of the mean. The right Y axis (red) represent different levels of glucose concentrations in (**A**) and (**B**). Arrows indicate sampling point for RNA isolation and dashed lines show the glucose level at that time point.

When GAS invades into deep tissue niches in the host, it can encounter an environment where the preferred carbohydrate, glucose, is not as readily available. Previous work found that C media accurately mimics this environment due to its initial low glucose, high peptide composition^35^. Here, we wanted to assess whether the presence or absence of glucose in the environment has an impact on the Mga virulence regulon of the M1T1 GAS 5448. The rich Todd-Hewitt Yeast (THY) media has an initial level of 0.5% glucose (w/v) and was used as a representative of a glucose-rich environment, while C media has a much lower initial level of 0.05% glucose (w/v) and was used to mimic a low- or no-glucose deep-tissue environment^35^. We directly assayed the change in the concentration of carbohydrates (primarily glucose) in THY (initially 240 mg/dl) and C media (initially 32 mg/dl) over an 8-h time course during GAS 5448 growth (Fig. 1). As expected, C media initially contained 8-fold less glucose than THY and it was expended earlier in log phase growth (Fig. 1). Total RNA was then isolated from four biological replicates from the WT 5448 grown in either THY or C media to late logarithmic phase (Fig. 1, arrows), the point of maximum Mga regulon expression^36^, and then processed for RNA-seq as described in Methods. At this point in growth, THY contained *ca*. 80 mg/dl glucose whereas all of the glucose had been consumed by GAS in C media for over 30 minutes (Fig. 1, dashed lines). RNA-Seq data generated from cells grown in THY was compared to C media-grown cells (THY/C media), where a 1.8-fold change in gene expression (log_2_ ≤ −0.90 or ≥0.90) and a *p* value of ≤0.05 were considered significant (Supplemental Table S1 and Fig. S1). Principal component analysis (PCA) of the RNA-seq data indicated reproducibility of the datasets from the same media, as well as a significant difference between WT 5448 grown in THY and C media (Supplemental Fig. S2).

**Figure 2:**
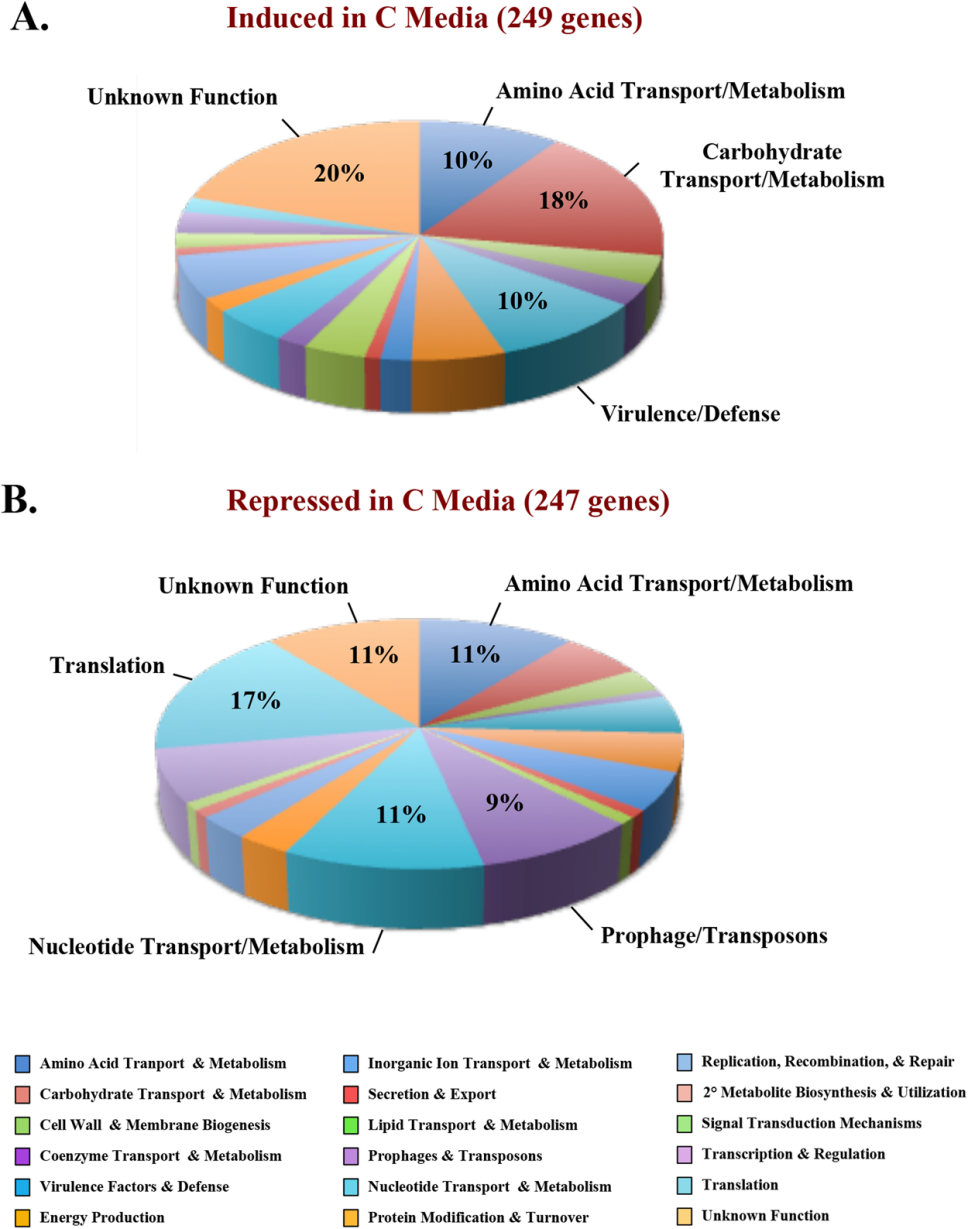
Functional categories of M1T1 GAS 5448 genes differentially expressed in C media versus THY. RNA-seq on M1T1 GAS 5448 grown in THY (high glucose) and C media (low glucose) to late logarithmic phase was analyzed for differential gene expression. GAS genes either induced (**A**) or repressed (**B**) in C media compared to THY were assessed based on their Cluster of Orthologous Group (COG) designation and displayed in pie graph format. The most prevalent COG designations are indicated with their overall percentage. Color scheme for COG categories are indicated at the bottom.

A total of 496 genes (26.9% of the non-tRNA genome) were found to be differentially expressed (249 induced, 247 repressed) in C media compared to THY (Fig. 2, Supplemental Table S1). Analysis of gene ontology using Cluster of Orthologous Groups (COGs) found that C media primarily induced the expression of genes encoding proteins involved in carbohydrate transport and metabolism (44 genes; 18% of induced genes), amino acid transport and metabolism (25 genes; 10% of induced genes), and proteins of Unknown Function (50 genes; 20% of induced genes) (Fig. 2A). In contrast, C media led to the down regulation of genes related to protein synthesis (43 genes; 17% of total repressed genes), amino acid transport and metabolism (26 genes; 11% of repressed genes), nucleotide transport and metabolism (26 genes; 11% of repressed genes), and proteins of Unknown Function (26 genes; 11% of repressed genes) (Fig. 2B). Induction of non-glucose carbohydrate and amino acid metabolism pathways in C media correlates with the composition of C media, which is high in peptides and low in glucose.

CcpA represses the expression of alternative carbohydrate utilization genes (*i*.*e*., CCR) through directly binding to *cre* sites under favorable glucose-replete conditions. Since C media contains no glucose at the time point taken, we predicted that CCR would be relieved. 119 (~24%) of the genes differentially expressed in C media (Supplemental Table S1) were also found to be regulated by CcpA in published transcriptome studies^17,18,37^. Of these, 59 had *cre* or *cre2* sites^37^ located upstream of genes that were either induced or repressed in C media (Supplemental Table S1), supporting that they could be directly regulated via CcpA. Thus, growth of WT 5448 in C media at late logarithmic phase partially resembles expression of a CcpA regulon in the absence of glucose (non-repressing condition)^17,18,37^.

In general, we found that most metabolic genes involved in utilization and transport of alternative (non-glucose) sugars were induced during late logarithmic phase growth in C media. These included many alternative PTS carbohydrate EII transporter genes such as those for 3-keto-L-gulonate (*ptxABC*), mannitol (*spy_1664*), galactose (*spy_1399*), both cellobiose operons (*spy_1079–1083* and *celB*), and trehalose (*treCB*). The ABC transporters for sialic acid *(spy_0213–0215*) and cyclomaltodextrin (*malADC*) were also upregulated. Interestingly, the β-glucoside-specific EIIABC and both mannose operons *ptsABCD* and *manLMN* were repressed in C media, suggesting they may represent sugar uptake systems for more preferred sugars (Supplemental Table S1).

Several virulence-related genes were upregulated during growth in C media, including the *has* (capsule synthesis) and *sag* (Streptolysin S) operons; which are also under CCR^17,18^. Although there is a considerable overlap between the C media and CcpA regulons, there were also media-specific regulatory phenotypes. An additional 25 genes found in this study have been previously shown to be regulated by CcpA^17,18^, but were regulated in a different direction. Interestingly, we observed a significant increase in the expression of the *lacD*.*1* operon and *rgg*/*ropB*, both of which encode for regulators of the secreted cysteine protease virulence factor gene *speB*. C media is an inducer of *speB* expression in M14 GAS^35^; however, we did not see *speB* being differentially expressed in M1T1 GAS 5448. We also found several genes of the core Mga virulence regulon to be down regulated in C media (Table 1) in comparison to THY, with the exception of *emm* and *mga*. Using real-time qPCR, we validated that *mga* and *emm* transcript levels were unaffected by growth in C media whereas the Mga-regulated genes encoding the fibronectin binding protein (*fba*) and the C5A peptidase (*scpA*) were down regulated.

**Table 1:** Expression of Mga core virulence regulon in THY compared to C media (THY/C).

Spy #	Annotation	Gene	RNA-Seq Log_2_ FC	qPCR Log_2_ FC
M5005_Spy1714	cell surface protein; Mga-regulated	*fba*	2.51	1.32 ± 0.17
M5005_Spy1715	C5A peptidase precursor; Mga-regulated	*scpA*	2.20	1.26 ± 0.33
M5005_Spy1716	Transposase		−1.66	—
M5005_Spy1717	Transposase		−2.47	—
M5005_Spy1718	inhibitor of complement; Mga-regulated	*sic1*.*0*	2.32	0.49 ± 0.14
M5005_Spy1719	M protein; Mga-regulated	*emm1*	0.74	0.84 ± 0.21
M5005_Spy1720	multi-virulence gene regulator Mga	*mga*	0.67	0.32 ± 0.11
M5005_Spy1721	hypothetical protein		−2.86	—

Taken together, the transcriptome of M1T1 GAS 5448 in C media exhibits similarity to the release of CcpA-mediated repression likely due to the absence of glucose in C media at late logarithmic phase. Additionally, expression of some of the Mga regulon, but not *mga* itself, is also reduced in C media compared to a glucose-rich environment.

### Comparison of the M1T1 GAS Mga regulon in THY to C media

**Figure 3:**
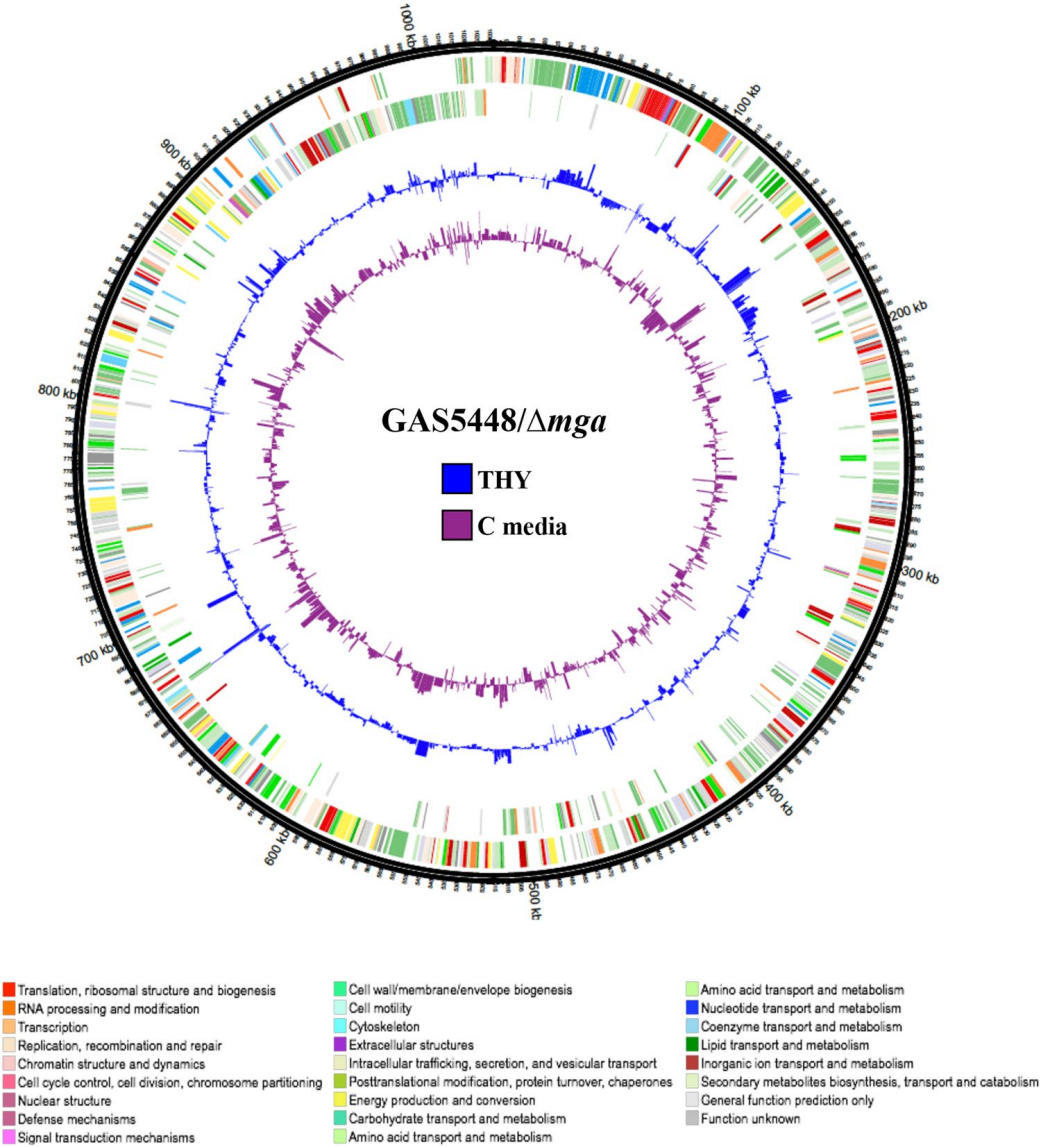
Transcriptomic landscape of M1T1 5448 ∆*mga* mutant during growth in low and high glucose. Circos plot of genes differentially expressed (DE) between GAS 5448 compared to an isogenic ∆*mga* 5448.930 grown in either C media (low glucose) or THY (high glucose) at late logarithmic growth. The outer most ring represents a size ruler and the next two rings represent the GAS open reading frames (ORFs) on the (+) and (−) strand of the genome, respectively. Color of ORFs reflect their functional Cluster of Orthologous Group (COG) as indicated at bottom. The inner rings show transcript levels (inside is down regulation, outside is up regulation) with the height of the bars representing the log_2_ fold-change in DE in THY (blue) vs. C media (purple).

Since we observed differential expression of Mga-regulated genes in different glucose concentrations without changes in *mga* itself, we hypothesized that the Mga regulon in these two medias would vary. An insertional inactivation of *mga* in M1T1 5448 was constructed using a temperature-sensitive plasmid to generate the *mga* mutant, 5448.930 (see Methods). Growth kinetics of 5448.930 were found to be comparable to WT 5448 in both THY and C media (data not shown). Total RNA was isolated in two biological replicates from the WT 5448 and the ∆*mga* mutant grown in either THY (+glucose) or C media (low glucose) to late logarithmic phase and processed for RNA-seq as described in Methods. Data obtained from the WT 5448 was compared to the ∆*mga* grown cells in either media (WT/∆*mga*) with changes in gene expression over 1.8-fold (log_2_ ≤ −0.90 or ≥0.90) and a *p* value of ≤0.05 considered significant (Fig. 3 and Supplemental Fig. S3).

The most activated genes in both media conditions were those of the core Mga regulon (*e*.*g*., *emm*, *sic*, *scpA*), showing between 7-fold to 18-fold reduction in transcript levels in the *∆mga* mutant (Table 2; Supplemental Figs S3 and S4**)**. Interestingly, we did not observe a Mga-specific phenotype for *sclA* in our RNA-Seq datasets, a gene considered to be part of the core Mga regulon^27,29,38,39^. The *mga* mutant 5448.930 was passaged at the permissive temperature to generate the 5448.930_R_ rescue strain as a complement control (Supplemental Fig. S3B). Real-time qPCR using the same RNA that was assayed by RNA-seq found that *sclA* was still regulated by Mga and it could be complemented in a strain 5448.930 R rescued for the *mga* mutation (Supplemental Figs S3AB and S4AB). This may result from a read alignment issue due to two *scl* alleles (*sclA*/*scl1* and *scl2*) encoded in the GAS genome, the second of which is not regulated by Mga.

**Table 2:** Genes regulated by Mga in both THY and C Media (WT 5448/*∆mga*).

Spy Number	Annotation	Gene	THY Log_2_ FC	C Media Log_2_ FC
M5005_Spy0113	transposase		1.47	1.32
M5005_Spy0143	hypothetical protein		2.54	3.35
M5005_Spy0522	unsaturated glucuronyl hydrolase		1.45	0.98
M5005_Spy0668	IgG-degrading protease	*mac*	1.62	1.37
M5005_Spy0824	tetrahydropholate synth., pyrophosphokinase	*folK*	−1.00	−0.87
M5005_Spy1076	putative bicarbonate transporter	*glnH*	−1.31	−0.85
M5005_Spy1077	putative bicarbonate transporter	*glnQ*.*2*	−1.04	−1.04
M5005_Spy1714	fibronectin-binding surface protein	***fba***	3.20	4.79
M5005_Spy1715	C5A peptidase precursor; Mga-regulated	***scpA***	3.43	5.04
M5005_Spy1718	secreted inhibitor of complement	***sic1***.***0***	4.63	5.53
M5005_Spy1719	M protein	***emm1***	9.08	10.06
M5005_Spy1720	multi-virulence gene regulator Mga	***mga***	4.60	5.84
M5005_Spy1721	hypothetical protein		−2.78	−5.00
M5005_Spy1733	hypothetical protein		−2.54	1.45
M5005_Spy1738	secreted DNase-Streptodornase-chromosomal	*spd*	0.98	1.03
M5005_Spy1798	suppressor of clpP/X, SpxA homolog, allele 2	*spxA2*	−1.86	−1.07

^*^Bold, shaded grey denotes the core Mga virulence regulon.

### M1T1 Mga regulon in THY (+glucose)

Published transcriptome studies of the Mga regulon from M1 SF370, M6 JRS4, M4 GA40634, and M59 MGAS15249 GAS strains at log phase in THY have indicated significant inter-serotype variation^27,29^. For RNA-seq of M1T1 5448 in THY, a total of 148 genes (excluding tRNA genes) were significantly regulated by Mga with approximately equal numbers of repressed (73) and activated (75) genes (Supplemental Table S2**;** Fig. 3, blue ring). Overall, 7.9% of the genome (excluding tRNA genes) was regulated by Mga in the M1T1 GAS 5448 background grown in THY to late logarithmic phase. To confirm the RNA-seq results generated from THY, 8 genes were chosen for analysis via qPCR, representing 6 activated and 2 repressed genes (Supplemental Fig. S3A). Real-time qPCR results validated the RNA-seq data with a correlation coefficient of 0.87 (Supplemental Fig. S3C).

Interestingly, the chromosomal *spd* gene, encoding the DNase Spd (Mitogenic Factor/MF, DNase B), exhibited 4-fold lower transcript in the mutant compared to WT 5448 (Supplemental Table S2), representing a new virulence gene activated by Mga. Mga repressed expression of the cysteine protease SpeB *(speB*) and its inhibitor (*spi*) in the M1T1 5448 background in THY (Supplemental Table S2, Fig. S3). This is in contrast to M1 SF370, where Mga activated *speB*^27^. Mga did regulate operons involved in sugar metabolism in M1T1 5448 comparable to M1 SF370^27^ (Supplemental Table S2). However, the majority of sugar-related operons were activated by Mga in 5448, the opposite of what was previously observed for SF370^27^. Therefore, despite similarities between many genes regulated by Mga in M1 SF370 and M1T1 5448, there were also clear differences in genes and/or directionality of regulation by Mga (Table 3). This further supported published evidence that the Mga regulon can vary across or even within serotypes grown under identical conditions.

**Table 3:** Comparison of the M1 SF370 and M1T1 5448 Mga regulons in THY.

MGAS5005	Gene Name	Annotation	Log_2_FC 5448 RNA-seq	SF370	Log_2_FC SF370 Microarray^*^
M5005_spy0143		hypothetical protein	2.54	SPy0169	1.95
M5005_spy0340	*lctO*	L-lactate oxidase	2.47	SPy0414	1.30
M5005_spy1632	*lacG*	6-phospho-beta-galactosidase	2.06	SPy1916	1.59
M5005_spy1714	*fba*	fibronectin-binding surface protein	3.20	SPy2009	5.59
M5005_spy1715	*scpA*	C5A peptidase	3.43	SPy2010	5.67
M5005_spy1718	*sic1*.*0*	secreted inhibitor of complement	4.63	SPy2016	6.13
M5005_spy1719	*emm1*	M protein	9.08	SPy2018	6.13
M5005_spy1720	*mga*	Multi-virulence gene regulator Mga	4.60	SPy2019	1.96

^*^Reference^27^.

### M1T1 Mga regulon in C media (low glucose)

Until now, the Mga regulon has only been described for GAS grown in THY^27,29^. RNA-seq analysis of the Mga regulon in C media at a point where glucose was consumed revealed a total of 135 genes (excluding tRNA genes) that were regulated by Mga (44 repressed, 91 activated) in C media, representing 7.2% of the GAS genome (Supplemental Table S3, Fig. 3; purple ring). Confirmation of 9 genes was assessed via real-time qPCR using RNA isolated from WT 5448, the *mga* mutant 5448.930, and its rescue strain (Supplemental Fig. S4), showing a correlation coefficient of 0.95.

In the absence of glucose, Mga activated expression of all three M1T1 DNases (streptodornases). As with THY, the chromosomally-encoded *spd* (MF, DNaseB) was also activated by Mga. In addition, the phage-encoded streptodornases *sdaD2* (Sda1) and *spd3* (Spd-3) were also regulated by Mga in C media, with both exhibiting *ca*. 5-fold reduction in a ∆*mga* mutant (Supplemental Table S3). Mga regulation of *sda2* in C media was confirmed by qPCR, showing an *ca*. 8-fold activation (Supplemental Fig. S4**;** green bar) that could be complemented upon glucose supplementation, indicating that Mga regulation of this streptodornase occurs in a glucose-dependent manner. In support of this finding, E64-treated (cysteine protease inhibitor) supernatants from *∆mga* mutants grown to late logarithmic phase in C media exhibited a decrease in steady-state Sda1 levels compared to WT 5448 (Supplemental Fig. S5) based on Western blots using polyclonal α-Sda1 antisera (gift from M. Walker).

Of note, we found a large number of virulence and metabolic genes to be Mga-regulated in C media that were not seen in the THY regulon. For example, genes encoding for Streptolysin S (*sagA*-*H*), Streptolysin O (*slo*), Streptokinase (*ska*), Streptococcal secreted esterase (*sse*), the heme utilization operon (*hupYZ*), and the osmotic stress operon (*opuAA*/*opuABC*) were regulated by Mga only in the glucose-depleted C media (Supplemental Table S3). Interestingly, Mga did not regulate *speB* in C media (Supplemental Fig. S4A, light blue bar), indicating that *speB* is potentially Mga-regulated in a glucose-specific manner in M1T1 GAS. Similarly, the known category B M1 Mga-regulated gene *grm*, encoding a 78-amino acid hypothetical cytosolic protein^21,27^, was found to be a part of the C media-specific regulon (Supplemental Table S3). Overall, we observed a broader induction of known virulence factors in a ∆*mga* background in C media, which appears to indicate that the presence or absence of glucose may impact the M1T1 GAS Mga regulon.

### Glucose levels alter the Mga regulon

**Figure 4:**
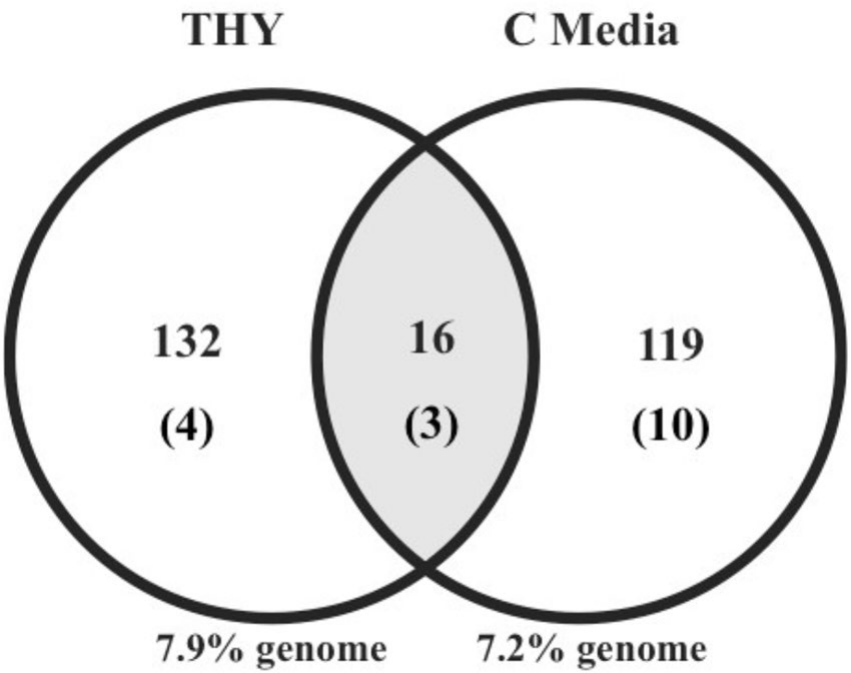
Comparison Mga regulons of GAS in high glucose THY and low glucose C Media. Venn diagram represents the numbers of Mga-regulated genes in either high glucose THY (left) or low glucose C media (right), with 16 genes found in common between the two data sets in the center (shaded). The number of tRNA genes found in each dataset (parentheses) are indicated, although percentage of regulation was calculated by excluding tRNA genes.

**Figure 5:**
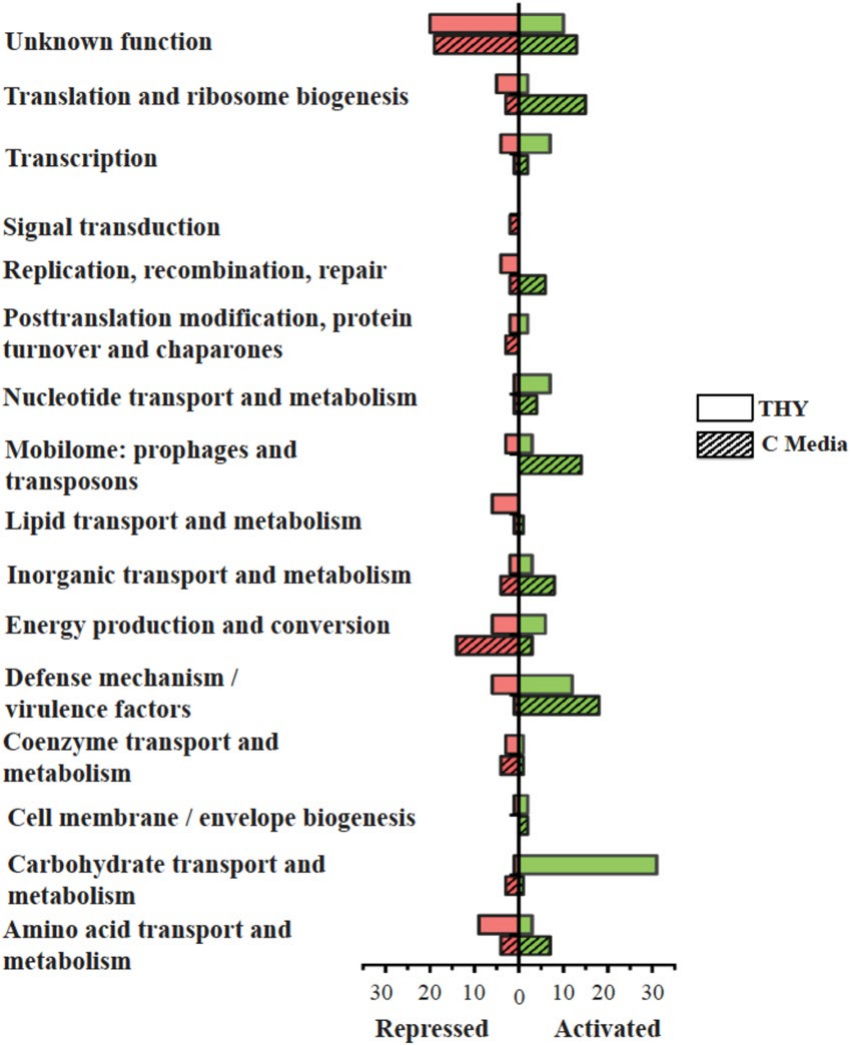
COG categories for Mga-regulated genes observed in THY and C media. A comparison of differentially expressed (DE) genes in various cluster of orthologous genes (COG) categories from THY (open bars) versus C media (crossed bars) of the GAS 5448 ∆*mga* 5448.930 mutant at late logarithmic growth when compared to the WT 5448. Bar length depicts the number of DE genes either down regulated (red) or up regulated (green) at *p* value of ≤0.05 with the total number of down and up regulated genes shown.

Comparison of the M1T1 5448 Mga regulon from THY and C media revealed significant differences in the genes that are Mga-regulated in the presence or absence of glucose (Table 2). Only 16 genes were found to be common between the two Mga regulons (Fig. 4; Table 2) comprising the core regulon virulence genes (Table 2; bold), as well as *glnH* and *glnQ*.*2* (bicarbonate transporter), *mac* (IgG-degrading protease), *spd* (chromosomally-encoded streptodornase), *M5005_spy0522* (sugar hydrolase), *M5005_ spy0143* (hypothetical protein), and *spxA*.*2* (a transcriptional regulator). Despite the stark difference in genes that are regulated by Mga in a glucose versus a no glucose condition, the broad COG categories represented were not different. In THY, the top three categories of Mga-regulated genes were unknown function (23 total), carbohydrate utilization (32 total), and defense mechanism/virulence (18 total) (Fig. 5). In C media, it was unknown function (32 total), defense mechanism/virulence (19 total), and energy production & conservation (17 total) (Fig. 5). This further supports that the PRD-containing Virulence Regulator (PCVR) Mga is a master regulator for genes involved in GAS pathogenesis and carbohydrate metabolism regardless of glucose levels.

Importantly, media-dependent Mga regulation was recapitulated using an independent *Krmit* transposon *mga* mutant KM16.1. Real-time qPCR was performed on selected targets from RNA isolated from WT 5448 and the KM16.1 *mga* mutant grown in either C media or THY. Four genes previously chosen for validation in THY (Supplemental Fig. S6A) and nine genes from C media (Supplemental Fig. S6B) were tested and identical media-dependent phenotypes in Mga-regulated gene regulation were seen for KM16.1 as in the 5448.930 mutant (Supplemental Fig. S3, S4 and S6).

### Supplementation of glucose in C media mimics a THY regulon

**Figure 6:**
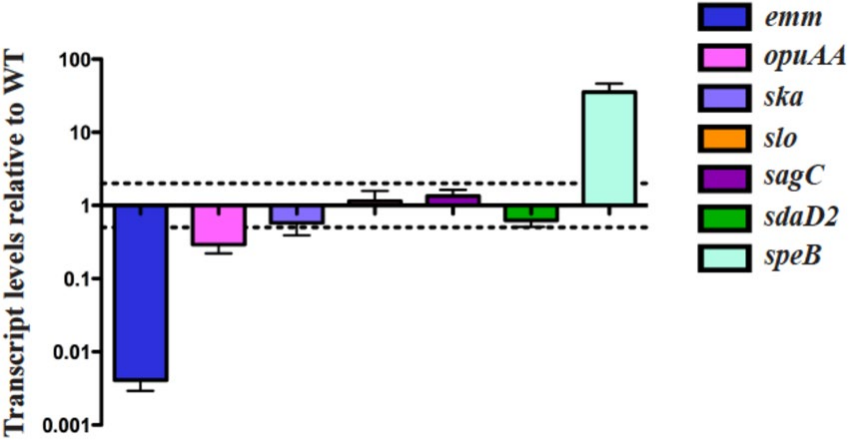
Supplementation of C media with glucose mimics some phenotypes observed for a ∆*mga* strain in THY. Transcript levels were determined using qPCR on RNA taken from WT GAS 5448 grown in C media supplemented to 0.5% glucose at late-log compared to ∆*mga* 5448.930 grown under the same conditions. Error bars represent the standard error from three biological replicates. Differences greater than 2-fold in expression for WT compared to mutant cells (dashed line) were considered significant.

Mga has an altered regulon in C media (low glucose) in comparison to THY (+glucose) (Fig. 3). We were interested in testing whether supplementing C media (32 mg/dl glucose) to a starting concentration comparable to THY (240 mg/dl) would mimic the THY regulon results. RNA was isolated for real-time qPCR from *∆mga* 5448.930 and the parental 5448 grown in C media supplemented to *ca*. 240 mg/dl glucose. A total of 5 differentially-regulated genes were chosen that were exclusive to the C media RNA-seq dataset (Supplemental Table S3; Fig. 6), as well as *speB*, which was only found to be regulated in THY (Fig. S6) and *emm*, which was observed in both datasets. We saw that 4 out of the 5 differentially regulated genes specific to C Media were no longer regulated by Mga when glucose was supplemented back to levels present in THY, including the phage-encoded DNase *sdaD2* (Fig. 6; green bar). Interestingly, *opuAA* (Fig. 6; pink bar) was the only gene that was not influenced by glucose.

Mga was shown to repress the transcription of *speB* in THY at late log phase (Supplemental Fig. S3A; light blue bar) and this phenotype was absent in C media (Supplemental Fig. S4; light blue bar). When glucose levels in C media were supplemented to levels found in THY, Mga was observed to repress *speB* again (Fig. 6; light blue bar). Therefore, while the mechanism by which Mga regulates *speB* is most likely indirect, there is a correlation between glucose availability and Mga regulation of *speB*. Overall, there appears to be two causes to the changes observed between THY and C media: (1) the majority of the changes appear to be specific to glucose availability, and (2) some changes in the regulon dynamics are due to C media-specific differences.

### Mga is differentially phosphorylated in C Media

**Figure 7:**
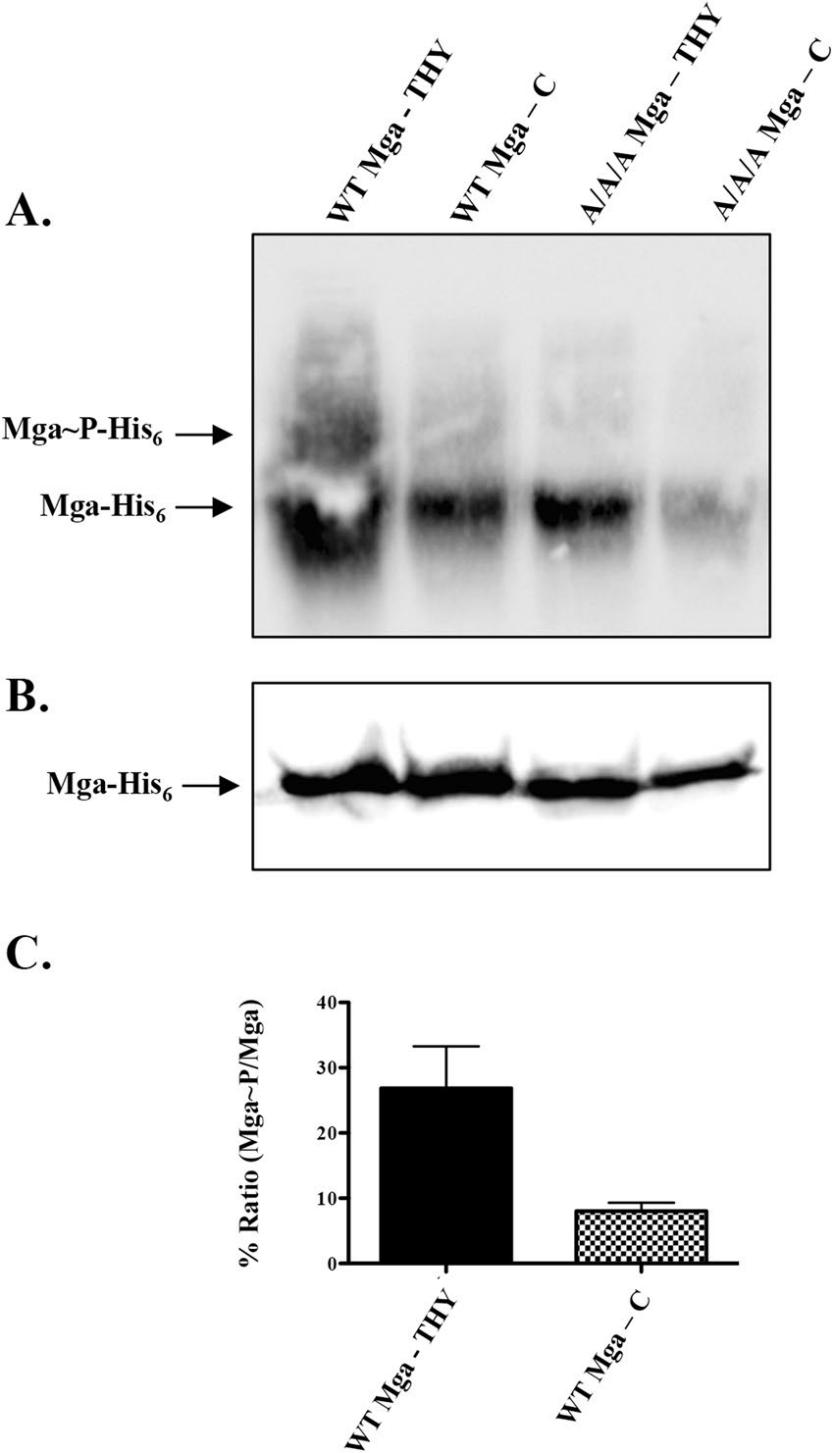
Mga is differentially phosphorylated based on glucose availability. The isogenic M4 *mga* mutant strain KSM547 complemented *in trans* with the His_6_- tagged WT strain (pKSM808) or phosphoablative A/A/A *mga* mutant (pKSM871) was grown in THY or C medium to the late exponential growth phase. Shown is a representative image of multiple replicates. (**A**) Equal concentrations of cell lysates of WT and A/A/A expressing strains grown in each media were resolved on a Phos-tag gel and immunoblotted using polyclonal αMga4 antibody. Bands corresponding to non-phosphorylated Mga and phosphorylated Mga are indicated by arrows. (**B**) Mga levels in same cell lysates were determined by 10% SDS-PAGE and immunoblotting using polyclonal αMga4 antibody. (**B**) Densitometric ratio of WT Mga~P-His_6_ bands to total Mga-His_6_ from THY and (**C**) Media lysates after separation on Phos-tag and Western blotting with a-Mga. Results were normalized to the same ratio in the respective A/A/A Mga-His_6_ control lanes from 2 biological replicates. Standard Error of the Mean (SEM) is indicated.

We observed differential expression of core Mga regulon genes of GAS 5448 grown in THY (+glucose) compared to C media (low glucose), yet *mga* itself was not affected (Table 1). Since Mga is a PRD-containing virulence regulator (PCVR) whose activity can be influenced by direct phosphorylation via the PTS in GAS, we asked whether the phosphorylation state of Mga was altered between these two environments. The Phos-tag gel matrix retards the migration of phosphorylated proteins and allows the identification of phosphorylation events via band shift compared to the non-phosphorylated protein of interest (see Methods). To study PTS-Mga interactions in GAS, we previously generated^3^ an M4 ∆*mga* (79% identity to M1 Mga) GAS strain (KSM547) that expressed a His-tagged version of either WT Mga (pKSM808) or a phosphoablative A/A/A Mga (pKSM871) that substitutes all three PTS-phosphorylatable histidines with alanines from a replicating plasmid (see Methods). The two strains were grown in either THY or C media to late log growth, whole cell lysates were isolated, and separated by Phos-tag SDS-PAGE for Western blot using α-Mga4 antibodies. The blot revealed two bands for WT 5448 lysates grown in THY, a slow-migrating more diffuse upper band arising from phosphorylated Mga at one or several of 3 potential histidines (Fig. 7A, Mga_4_~P-His_6_) and a larger fast-migrating band corresponding to non-phosphorylated Mga (Fig. 7A, Mga_4_-His_6_). In C media at a point where glucose is depleted, the slow-migrating Mga_4_~P-His_6_ is reduced, indicating less PTS phosphorylation (Fig. 7A). As would be expected, the Mga_4_~P-His_6_ band is absent in the phosphoablative A/A/A M4 Mga control mutant regardless of the media (Fig. 7A). The ratio of Mga_4_~P-His_6_ compared to total Mga_4_-His_6_ in the two WT lanes from two biological replicates was determined by densitometry analysis and normalized to the same ratio in the respective A/A/A Mga lanes for each (Fig. 7C). This showed a 3-fold increase in PTS-dependent phospho-Mga in THY compared to C media. As a control for loading, the same cell lysates were subjected to Western blot after SDS-PAGE using α-Mga4 antibodies (Fig. 7B). While a comparable amount of protein was detected for all strains in the Western blot, we consistently observed less protein for the A/A/A M4 Mga mutant grown in C media and assessed by Phos-tag and this phenomenon is currently being explored further. Together, these data provide the first evidence that Mga can be alternatively phosphorylated in the presence or absence of glucose.

## Discussion

The current study was undertaken to assess how glucose affects global transcriptional changes in the GAS cell through Mga. Our RNA-seq results revealed that in the WT M1T1 5448 some of the Mga regulon is up regulated in THY (+glucose), but not *emm* and *mga* itself. There was a strong correlation between the genes activated in C media (low glucose) with those known to be repressed by CcpA, indicating that C media relieves CCR. Interestingly, when comparing the Mga regulon in both medias, we found very little overlap beyond the core virulence genes, despite regulating similar functional categories (COGs). Many of these differences in Mga-regulated genes between the two medias could be directly attributed to glucose levels, which led to an alteration in the PRD-mediated phosphorylation patterns of Mga. Thus, Mga and its regulon are likely influenced by glucose and further support an *in vivo* link between glucose availability and virulence regulation in GAS.

### The C media transcriptome of M1T1 GAS

For low G + C Gram-positive bacteria, carbon catabolite repression (CCR) is controlled by the master regulator, CcpA, through direct binding to catabolite response elements (*cre*) sites. Metabolic genes that enable the utilization of alternative (non-glucose) sugars are classically induced in the absence of the preferred carbohydrate source, glucose. For GAS, two studies have determined the genome-wide, CcpA-induced regulons in M1T1 serotype^17,18^, which found that ~8% of the GAS genome was regulated by CcpA in THY. Here, we observed that 16% of the GAS genome was differentially expressed when grown in the absence of glucose (C media at late log phase), which overlapped with the published CcpA regulons^17,18^. Thus, it appears that the lack of glucose encountered by GAS in C media at this point in growth is capable of relieving CCR and stimulating the transcription of alternative carbohydrate uptake systems. Although there are clear commonalities, our data also showed that GAS undergoes a much broader transcriptomic change during growth in C media (476 genes differentially expressed, 25.5% of genome) when compared to growth in THY (Supplemental Table S1). Therefore, growth in C media mimics a low- to no-glucose environment, but it also creates other changes in the cell that are likely attributed to high levels of peptides present in C media.

The C media-induced regulon was established for the serotype M14 HSC5 using altered buffered and osmotic conditions at early stationary growth phase^35^. The authors found C media to be a representative of conditions encountered by GAS during deep tissue infection due to up regulation of the virulence factor and cysteine protease gene, *speB*, to levels comparable to those observed during *in vivo* infection^35^. Thus, C media has been used in several studies for optimal expression of *speB*. Surprisingly, we did not observe a significant induction of *speB* in C media. However, the current study was conducted in late logarithmic phase, and not stationary phase, where *speB* is known to be most highly expressed. We did observe an increase in expression of both *lacD*.*1* and *rgg*/*ropB*, which have been shown to interact with and stimulate *speB* expression^40^. A previous study found that *in vivo*/environmental signals suppress the expression of *speB*, and induce the expression of *speA*^41^. However, we saw no observable change in *speA* in our RNA-Seq dataset of 5448 grown in THY compared to C media. Therefore, the conditions tested here may not be optimal for the induction of *speB* expression in M1T1 5448.

### Plasticity of the M1T1 Mga virulence regulon under varying glucose levels

The Mga regulon was initially determined by our group based on microarray analysis of 3 diverse serotypes representing both *mga-1* and *mga-2* alleles (M1, M6 and M4) grown to late-logarithmic growth in THY, involving ~10% of the GAS genome that encompassed mostly virulence and carbohydrate metabolism genes^27^. Recently, RNA-seq of Mga-regulated genes in an M59 (*mga-2*) serotype, also at late-logarithmic growth in THY, revealed ~7% of the genome was involved, primarily related to virulence, metabolism, and unknown functions^29^. Our RNA-seq results revealed that the Mga regulon in an M1T1 GAS strain under similar growth conditions regulated ~8% of the genome, in line with the recent M59 regulon. Although the M1 (SF370) and M1T1 (5448) strains analyzed differ by only 7% in their genomes (mostly prophage content), comparison of the Mga (*mga-1* allele) regulon datasets revealed very little overlap. A similar trend was observed when regulons from strains expressing two *mga-2* alleles (M59 and M4) were compared^29^. We saw that SF370 and 5448 shared only 8 Mga-regulated genes that were regulated in the same direction, with 5 genes being members of the core Mga-regulated virulence genes (Table 3). Thus, a common theme amongst all Mga regulons is the activation of core virulence genes when present (*e*.*g*., *emm*, *sic*, *fba*, *scpA*, *sclA*, *sof*), although the degree of activation appears to vary^27,29^.

The other three Mga-regulated genes shared between M1 and M1T1 GAS were *lacG* (6-phospho-beta-galactosidase), *lctO* (lactate oxidase), and *M5005_spy0143* (hypothetical protein). Both *lacG* and *lctO* are involved in lactose metabolism. LctO converts lactate into pyruvate and leads to production of hydrogen peroxide (H_2_O_2_) in a growth phase- and glucose-dependent manner through direct repression by CcpA^19^. In *S*. *pneumoniae*, increase of H_2_O_2_ production acts as a virulence trait that aids in colonization by slowing the clearance of bacteria through the inhibition of ciliary beating in the upper respiratory tract and by promoting bactericidal activity to outcompete other microbial species from the microflora^42–44^. Therefore, it’s possible that Mga activates the transcription of *lctO* in order to up regulate hydrogen peroxide production just enough to aid in colonization, which is the stage of infection that correlates with the highest level of Mga activation^15^. Interestingly, *M5005_spy0143* encoding a small hypothetical protein was also found to be regulated in C media (Table 2 and Supplemental Table S3), and was the only gene other than the core regulon found in common across the different serotypes and medias.

The THY Mga regulon of M1T1 5448 showed activation of several sugar-specific operons, in contrast to the repression previously observed in the published regulons^27,29^. M1T1 Mga appears to regulate carbon metabolism genes comparable to what was observed for MafR, a recently characterized Mga-homolog and PCVR implicated in the pathogenesis of *E*. *faecalis*^45^. Further, the cysteine protease, *speB*, and the SpeB inhibitor (*speI*) were both observed as being repressed by Mga in M1T1 5448 grown in THY (Supplemental Table S2 and Fig. S3, light blue bar) whereas *speB* was previously shown to be activated by Mga in a serotype-specific manner^27^. Interestingly, no change in the transcript of *ropB*/*rgg* or *lacD*.*1* was observed in the 5448 ∆*mga* mutant, both of which are known to regulate *speB* expression^16,40^. Given that regulation of *speB* by Mga was restored when glucose was added back to C media (Fig. 6), there appears to be a glucose-dependent Mga regulation of *speB* in the M1T1 background. Regardless of the differences, it is apparent that Mga regulates a variety of genes important for colonization and, albeit indirect, metabolic operons in the presence of glucose.

In both THY and C media, Mga regulates the same approximate number (155 versus 148 genes, respectively) of the non-prophage GAS genome and representing comparable COG categories (Figs 4 and 5). Yet, there was a stark contrast in the genes represented between the datasets from the two medias. A total of only 16 genes (~10%) overlapped between THY and C media, half of which are established members of the core Mga virulence regulon (Table 2) and the remaining 8 genes being unique to the M1T1 5448 Mga regulon. Of these, *glnH* and *glnQ* encode a putative bicarbonate transporter. Mga is activated under elevated CO_2_ conditions^46^, and this environmental signal may act independently of other activation signals, as is seen for the *B*. *anthracis* PCVR AtxA and the group G Streptococcus (GBS) DmgB, both of which are homologs of Mga^24^. Therefore, a potential link between bicarbonate transport and Mga gene regulation may exist. Since the number of genes that overlap between the THY and C media Mga regulon are so few, we propose that this set of nine novel genes may also be a part of the core M1T1 Mga regulon.

### Streptodornases represent novel Mga-regulated virulence genes

Both M1T1 phage-encoded DNases (*sda1*and *spd3*) were activated by Mga in C media (Supplemental Table S3) whereas the chromosomally-encoded *spd* was activated in both the presence and absence of glucose (Table 2). In M49 NZ131, the stand-alone regulator Rgg was found to bind directly to the upstream region of *spd3*, and directly repress transcription of this phage-encoded nuclease^47,48^. We also demonstrated that Mga regulates expression of *sdaD2* in a glucose-dependent manner (Figs 6 and 7). Interestingly, *sdaD2* is one of two horizontally transferred genes that distinguishes the invasive M1T1 serotype^49^ and has been suggested to provide selective pressure for the *covS* switch in the M1T1 5448 (*covS*^+^) *in vivo*^50,51^. It has been suggested that in the *covS*^+^ M1T1 MGAS2221, the Mga regulon, not Sda1, plays a much more important role in the *covS* switch *in vivo*^52^. It may be that during invasive infection with *covS*^+^ M1T1 strains, a glucose-deplete environment leads to Mga-dependent activation of *sdaD2* (and other streptodornases) that impacts the *covS* switch.

### Glucose levels affect Mga phosphorylation and activation of its regulon

We observed a 4- to 5-fold repression of some core Mga regulon genes (*fba*, *scpA*, *sic*, etc.) in C Media (Table 1) in the WT 5448; however, a significant change in transcription of *emm* was not observed. Two possibilities for the reduction of Mga regulon expression in C media compared to THY are: (1) CcpA has been shown to bind to a *cre* site located upstream of the P1 promoter region of *mga* and stimulates transcription^53^; therefore, in the low-glucose environment of C media, CCR is relieved, causing a decrease in expression of the Mga regulon under a low glucose condition. Alternatively, (2) PTS-mediated phosphorylation of Mga activity may impact regulation of the core genes^3,29^. The latter scenario is the most probable, as the transcript levels of *mga* were not significantly different between THY and C media, indicating possible posttranslational modification. The lack of differential *emm* expression is not clear at this time and we are exploring this mechanism further.

The differential phosphorylation patterns of Mga observed using Phos-tag supports that PTS involvement can affect the transcription of the regulon in one of three ways: (1) no phosphorylation produces an active Mga, (2) phosphorylation on only PRD2 can cause an increase in activity, and (3) phosphorylation on PRD1 inhibits Mga activity, and is dominant over PRD2 phosphorylation^3^. Therefore, we hypothesize that the low- to no-glucose environment encountered in C media would likely cause phosphorylation on PRD1, causing a reduction in Mga activity and regulon expression, whereas growth in THY (high glucose) would result in an active non-phosphorylated Mga and remain active. Hence this would help explain the activation of the regulon in THY over C media (Table 1). Furthermore, a phosphoablative Mga mutant would eliminate the PTS phosphorylation phenotype regardless of media.

Surprisingly, Mga from GAS grown in THY showed two bands on a Phos-tag gel representing a phosphorylated and unphosphorylated species. In C media, far less of the phosphorylated form of Mga was observed, while the phosphoablative (A/A/A) construct was unphosphorylated under both media conditions (Fig. 7). Therefore, we now hypothesize that in C media, when glucose is depleted, Mga still may be in its “active” form (condition 1), and THY represents a “hyper-active” form of Mga (condition (2). This would be in line with the model that was previously proposed by Hondorp *et al*.^3^, since glucose is initially present in both THY and C media. Further, since RNA was isolated from strains grown to late log, there still may be a small but significant amount of glucose present in C media to maintain the phosphorylation status of Mga. Regardless, we now believe it may not only be the level of glucose that is important for Mga activity, but the mere presence of it during growth which could affect the phosphorylation status of Mga. The unphosphorylated band for the A/A/A Mga in both C media and THY indicates that phosphorylation by the PTS occurs at the phosphohistidines on the PRD domains of Mga.

#### Concluding Remarks

This work shows that glucose directly affects Mga activity and the composition of the Mga regulon. Furthermore, the differences between the regulons in the presence or absence of glucose is most likely attributed to a phosphorylation event that occurs on the phosphohistidines located on the PRD domains of Mga. While it is unclear which phosphohistdine(s) are affected and the mechanism by which Mga is able to regulate genes alternatively, it is clear that in the presence of glucose, the PTS plays an important role in activating this global transcriptional regulator and virulence genes downstream.

## Methods

### Bacterial Strains and Media

*Streptococcus pyogenes* (GAS) strain M1T1 5448^54^ is isolated from an invasive infection. The reference genome used in this study was generated from the M1T1 strain MGAS5005^55^. GA40634 is a clinical isolate of the GAS M4 serotype and SF370 is a sequenced M1 strain. KSM547 and KSM165-L are isogenic strains of GA40634 and SF370, respectively, with an insertional inactivation of the *mga* gene^27,38^. GAS bacteria were either cultured in Todd-Hewitt medium supplemented with 0.2% yeast extract (THY) or in C Media^56^ buffered to pH 7.5 with NaOH. A 25% (w/v) stock solution of glucose was used to supplement C media to the levels indicated in select experiments. GAS growth was assayed via absorbance using a Klett-Summerson colorimeter (A filter) and expressed in Klett Units.

*Escherichia coli* (*E*. *coli*) strain DH5α (*hsdR17 recA1 gyrA endA1 relA1*) and C43[DE]^57^ were used as the host for plasmid constructions, and C41[DE3]^57^ was used for protein expression, as indicated in Table 4. *E*. *coli* strains were grown in Luria-Bertani (LB) broth or ZYP-5052^58^ for protein expression. Antibiotics were added to media, as needed, at the following concentrations: spectinomycin at 100 µg/ml for both *E*. *coli* and GAS, kanamycin at 50 µg/ml for *E*. *coli* and 300 µg/ml for GAS, and 100 µg/ml of ampicillin for *E*. *coli*.

**Table 4:** Bacterial strains and plasmids used in this study.

Strain/Plasmid	Relevant genotype	Reference
**Strains**		
*E*. *coli*		
DH5α	*hsdR17 recA1 gyrA endA1 relA1*	^72^
C41[DE3]	*F*^*-*^ *ompT gal dcm hsdS*_*B*_ (r_B_^−^m_B_^−^)(DE3)	^57^
C43[DE3]	*F*^*-*^ *ompT gal dcm hsdS*_*B*_ (r_B_^−^m_B_^−^)(DE3)	^57^
*S*. *pyogenes*		
5448	M1T1	^54^
5448.930	∆*mga* mutant in 5448 (insertional inactivation)	This study
5448.930 _R_	*mga* + 5448, cured strain	This study
KM16.1	∆*mga* mutant in 5448 (transposon)	This study
GA40634	M4 GAS, clinical isolate	^73^
KSM547	∆*mga* mutant in GA40634	^38^
SF370	M1 GAS	^74^
KSM165L	∆*mga* mutant in SF370	^27^
**Plasmids**		
pCRS	Temperature-sensitive conditional vector; Sp^R^	^23^
pJRS525	Replicating vector for GAS with Sp^R^	^36^
pKSM807	WT *mga1* under native P*mga1*	^34^
pKSM808	WT *mga4-his6* (GA40634) under native P*mga4*	^34^
pKSM809	WT *mga1-his6* under native P*mga1*	^34^
pKSM871	H204A/H270A/H324A *mga4*-*his6* under native P*mga4*	^3^
pKSM930	pCRS construct; insertional inactivation of *mga*	This study
pLZ12-Spec	Broad host range cloning vector, Sp^R^	^75^
pMga1-His	WT *mga1* (SF370) with C-terminal His6	^34^
pUC19	ColE1 ori Ap^R^ *lacZ*α	^76^

### DNA manipulations

Plasmid DNA was extracted from *E*. *coli* using the Wizard Plus SV miniprep system (Promega). DNA fragments were agarose gel purified using the Wizard SV gel and PCR cleanup system (Promega). PCR for generating probes and cloning was conducted using Accuprime Pfx (Life Technologies) according to the manufacturer’s recommended protocol. PCR for diagnostic assays was performed using Taq DNA polymerase (NEB). Genewiz, Inc performed all DNA sequencing. The Master-Pure complete DNA and RNA purification kit for Gram-positive bacteria (Epicentre, Illumina) was used to extract genomic DNA.

### Insertional inactivation and rescue of GAS 5448 *mga* mutants

An insertional inactivation construct was generated using a 300 bp PCR internal fragment of *mga* from 5448 gDNA, which was amplified using the primers Mga InIn F and Mga InIn R (Supplemental Table S4). The fragment was cloned via *BamHI* sites into pCRS, generating pKSM930 (Table 4). GAS 5448 was transformed with 20 µg of plasmid (pKSM930) and grown on THY agar containing spectinomycin at 30 °C. Potential integration *mga* mutants (GAS 5448.930) were identified following selection with spectinomycin and growth at the non-permissive temperature (37 °C). PCR on isolated gDNA was used to confirm plasmid insertion for each GAS mutant using primers SPR1 and SPR2 (Supplemental Table S4).

The strain 5448.930 (Table 4) was cured of the plasmid inactivating *mga* by passage in liquid culture four times at 30 °C with no drug selection. Cultures were then plated, and patched on THY supplemented with spectinomycin and THY alone. Patches that showed spectinomycin sensitivity were then screened for loss of the spectinomycin gene by PCR using the primer SPR1 and SPR 2 (Supplemental Table S4).

A transposon insertion in *mga* was identified from a pool of *Krmit* (for kanamycin-resistant transposon for massive identification of transposants) transposon mutants generated in 5448^59^. Library #16 was screened using AP-PCR^60^ followed by sequencing and alignment of sequence to the 5448 genome. Transposant #1 (16.1) was mapped to the 5′-end of *mga* in the sense direction.

### RNA-Seq and data analysis

RNA sequencing (RNA-Seq) was performed as previously described^21^. Briefly, Direct-zol RNA MiniPrep kit (Zymo Research) was used to isolate total RNA using a modified procedure to improve GAS cell disruption. Frozen cell pellets were resuspended in 700 µl of Trizol plus 300 mg of acid-washed glass beads (Sigma Life Science) and disrupted by vortexing for 5 min. RNA samples were DNase-treated using the Turbo DNase-free kit (Life Technologies). A total of 5 µg of this RNA was treated for ribosomal RNA (rRNA) removal using the Ribo-Zero Magnetic kit for Gram-positive bacteria (Epicentre). Quality and quantity were assessed using a 2100 Bioanalyzer (Agilent) NanoDrop 8000 spectrophotometer (Thermo Scientific), respectively. Directional RNA-Seq libraries were created using the ScriptSeq v2 RNA-Seq Library Preparation kit (Illumina) according to the manufacturer’s recommendations.

A rapid-run 100 bp single-read DNA sequencing was then performed at the Institute for Bioscience and Biotechnology Research (IBBR) Sequencing Facility at the University of Maryland, College Park, using the Illumina HiSeq 1500 platform. Data were generated in the standard Sanger FastQ format and raw reads were deposited with the Sequence Read Archive (SRA) at the National Center for Biotechnology Institute (accession PRJN412519).

Read quality was measured using FastQC^61^, filtered and trimmed using trimmomatic^62^ and mapped against the MGAS5005 genome (accession CP000017) using alignment software, as previously described. Differential expression analyses were performed following size-factor and quantile normalization of read counts. Limma^63^ and DESeq^64^ statistical models were used to account for batch effects. The resulting metrics of expression were visualized using Circos^65^, and tested for ontology enrichment using a variety of ontology software packages (KEGG^66^, goseq^67^, clusterProfiler^68^, GOstats^69^, and topGO^70^).

### qRT-PCR

The quantitative (real-time) reverse transcription-PCR (qRT-PCR) experiments were performed as previously described^21^. Briefly, DNase-treated total RNA from strains were added to SYBR green master mix (Applied Biosystems) with 6.5 µl of each gene-specific real-time primer from a 20 nM stock (Table 2) using the one-step protocol on a Light Cycler 480 (Roche). Real-time primers were designed using the interactive tool Primer3 (http://biotools.umassmed.edu/bioapps/primer3_www.cgi).

RNA-Seq data validation was performed as previously described^21^, using cDNA generated separately in a two-step protocol in order to take into account the strand-specificity of the RNA-Seq results.

All qPCR results are depicted as ratios of the experimental/wild type levels relative to *gyrA* transcripts, which acts as an internal control. Standard error was calculated from three biological replicates, and differences over 2-fold in expression were considered significant. Correlation coefficients were determined by graphing the log value of the RNA-seq result on the X-axis to the log value of the RT-qPCR on the Y-axis. The R^2^ value was calculated using a linear regression model, which represented the fitness of the data (Supplemental Fig. S1).

### Glucose utilization monitored by Glucose Test Strips

Glucose utilization was monitored using a blood glucometer (AimStrip® Plus). Although typically used to monitor blood sugar, we used this apparatus to monitor glucose concentration of THY, CDM, and whole human blood during GAS growth. Values are given in mg/dl. Readings were taken by first inserting a test strip (AimStrip® Plus) into the monitor, placing the test strip into the culture to draw up liquid droplets, and waiting for 10 seconds for the final reading.

### Mga-1 purification and expression

GAS Mga1-His_6_ was purified as previously described^34^. Briefly, *E*. *coli* containing the plasmid pMga1-His was grown in ZYP auto-induction media for ~50 h at 37 °C. Cells were harvested by centrifugation at 4 °C. Pellets were then resuspended in Lysis Buffer A (20 mM NaP_i_, 500 mM NaCl, 20 mM imidazole, pH 7.4) and 1× EDTA-free cOmplete protease inhibitor (Roche), and incubated on ice with lysozyme for 30 min followed by sonication using a Branson Sonifier 450 with a tapered microtip (setting 6, 50% duty cycle) pulsing 3 × 1 min with 3 min breaks on ice between cycles. The lysate was spun for clarification at 20,000 × *g* for 30 min at 4 °C 3–4 times. The lysate was loaded on a 5 ml NiNTA agarose resin (Qiagen) and rotated at 4 °C for 1 h. The column was then washed twice with NiNTA Wash Buffer (50 mM NaH_2_PO_4_, 300 mM NaCl, 20 mM imidazole, pH 8.0). Protein was eluted with 10 ml of NiNTA elution buffer (50 mM NaH_2_PO_4_, 300 mM NaCl, 250 mM imidazole, pH 8.0) and chosen fractions were stored at −20 °C. Protein concentration was analyzed using the Bio-Rad protein assay kit, reading the absorbance at 595 nm on a spectrophotometer.

### GAS whole cell lysates

Soluble GAS protein extractions were performed using the bacteriophage lysine, PlyC (kindly provided by D. Nelson) as previously described^34^. Briefly, GAS cells were inoculated 1:20 into 12 ml of THY or C media^56^, grown to late-log, and pelleted by centrifugation for 15 min at 6,000 × *g*. Pellets were resuspended in 200 µl of Buffer D (30 mM Tris pH 7.5, 0.1 mM dithiothreitol [DTT], 40% (v/v) glycerol), 1 × cOmplete EDTA-free protease inhibitor (Roche), 10 µl (20 U) of Turbo DNase (Life Technologies), and 5 µl (250 U) of PlyC^71^. The cells were mixed by flicking and incubated on ice for 20 min, followed by centrifugation at 13,000 × *g* for 10 min at 4 °C. Clarified supernatants containing soluble proteins were extracted and boiled with 3 × cracking buffer (0.15% [w/v] Bromophenol Blue, 0.6% [w/v] SDS, 3 ml glycerol, 3.9 ml Tris-HCl [500 mM, pH 6.8], 1.5 ml β-mercaptoethanol, and 4 ml dH_2_O) at 95 °C for 5 min, and stored at −20 °C. Protein concentration was assayed using the Bio-Rad protein assay kit, reading the absorbance at 595 nm on a spectrophotometer.

### Western Blots

Selected protein samples were run on a 10% SDS-PAGE, with 6% stacking gel, for 1 h at 180 V. The gels were then transferred to nitrocellulose membranes using the Mini-Protean apparatus (Bio-Rad) in 1X transfer buffer (25 mM Tris base, 0.2 M glycine, 20% methanol). Membranes were blocked overnight at 4 °C in blocking solution (5% (w/v) dried milk in PBS-tween). Primary antibodies were incubated at a 1:1,000 dilution, unless otherwise stated. The following primary antibodies were used at a 1:1,000 dilution: α-His antibody (Roche), α-Mga4, α-Mga1-HRP, and α-Sda1 (kindly provided by M. Walker). Blots were then washed three times for 5 min in PBS-tween. The primary antibody Hsp60 (Enzo Life Sciences) was used at a 1:2,500 dilution. Blots were then incubated with secondary antibodies: α-rabbit-HRP (1:5,000) or α-mouse-HRP (1:20,000) for 1 h. The blots were then washed with PBS-tween three times for 5 min, and visualized using SuperSignal West Femto Substrate (Thermo Scientific) and a LAS-3000 CCD camera (FUJIFILM).

### Phos-tag phosphate affinity gel

The phos-tag gel system was used to visualize the phosphorylation state of Mga. Briefly, GAS whole cell lysates were run on a Mn^2+^-Phos-tag 10% SDS-PAGE gel (WAKO), with 6% stacking gel. The resolving gel was made as follows: 1.75 ml of 1.5 M Tris-HCl, 1.75 ml of 40% bis-acrylamide, 140 µl of 10 mM MnCl, 140 µl of phos-tag solution, 3.1 ml of dH_2_O, 100 µl of 10% ammonium persulfate, and 20 µl of TEMED (Bio-Rad). Protein samples were run for 2 h at room temperature and 2 h on ice, for a total of 4 h at 180 V, prior to western blot as described above.

### Data Availability Statement

The authors have deposited all RNA-seq raw sequencing reads with the Sequence Read Archive (SRA) at the National Center for Biotechnology Institute (accession PRJN412519) for public availability.

## Electronic supplementary material

Supplementary Materials
