# Supplementary material for: Glucose Levels Alter the Mga Virulence Regulon in the Group A Streptococcus
Source: Sci Rep. 2018 Mar 21;8:4971. doi: 10.1038/s41598-018-23366-7 (PMC5862849; doi:10.1038/s41598-018-23366-7)
Supplement: Supplementary file 1 — Supplementary Materials [file 41598_2018_23366_MOESM1_ESM.pdf]

Supplementary Materials for:

Glucose Levels Alter the Mga Virulence Regulon in the Group A Streptococcus

Kayla M. Valdes[1], Ganesh S. Sundar[1], Ashton T. Belew[2], Emrul Islam[1], Najib M. El-Sayed[1,2], Yoann Le Breton[1], and Kevin S. McIver[1]

[1]Department of Cell Biology & Molecular Genetics and Maryland Pathogen Research Institute, University of Maryland, College Park (UMCP), College Park, Maryland, USA; [2]Center for Bioinformatics and Computation Biology, UMCP, MD USA

Supplementary Materials Contents:

Supplemental Figures. Following six (6) figures are included:

Figure S1. Transcriptomic landscape of M1T1 5448 during growth in C media compared to THY.

Figure S2. Clustering of WT 5448 samples based on RNA-seq data.

Figure S3. Confirmation of THY RNA-Seq results for Mga regulon using qPCR.

Figure S4. Confirmation of C media RNA-Seq results for Mga regulon using qPCR.

Figure S5. Sda1 levels are reduced in Δ*mga* M1T1 GAS supernatants grown in C media.

Figure S6. Media-dependent transcriptional regulation by Mga is recapitulated in an independent 5448 Δ*mga* mutant.

Supplemental Tables. Following three (3) tables are included:

Table S1. Genes in 5448 differentially expressed (DE) in THY compared to C Media

Table S2. RNA-Seq results of the *mga* mutant in THY (WT/Δ*mga*)

Table S3. RNA-Seq results of the *mga* mutant in C Media (WT/Δ*mga*)

Table S4. PCR primers used in this study

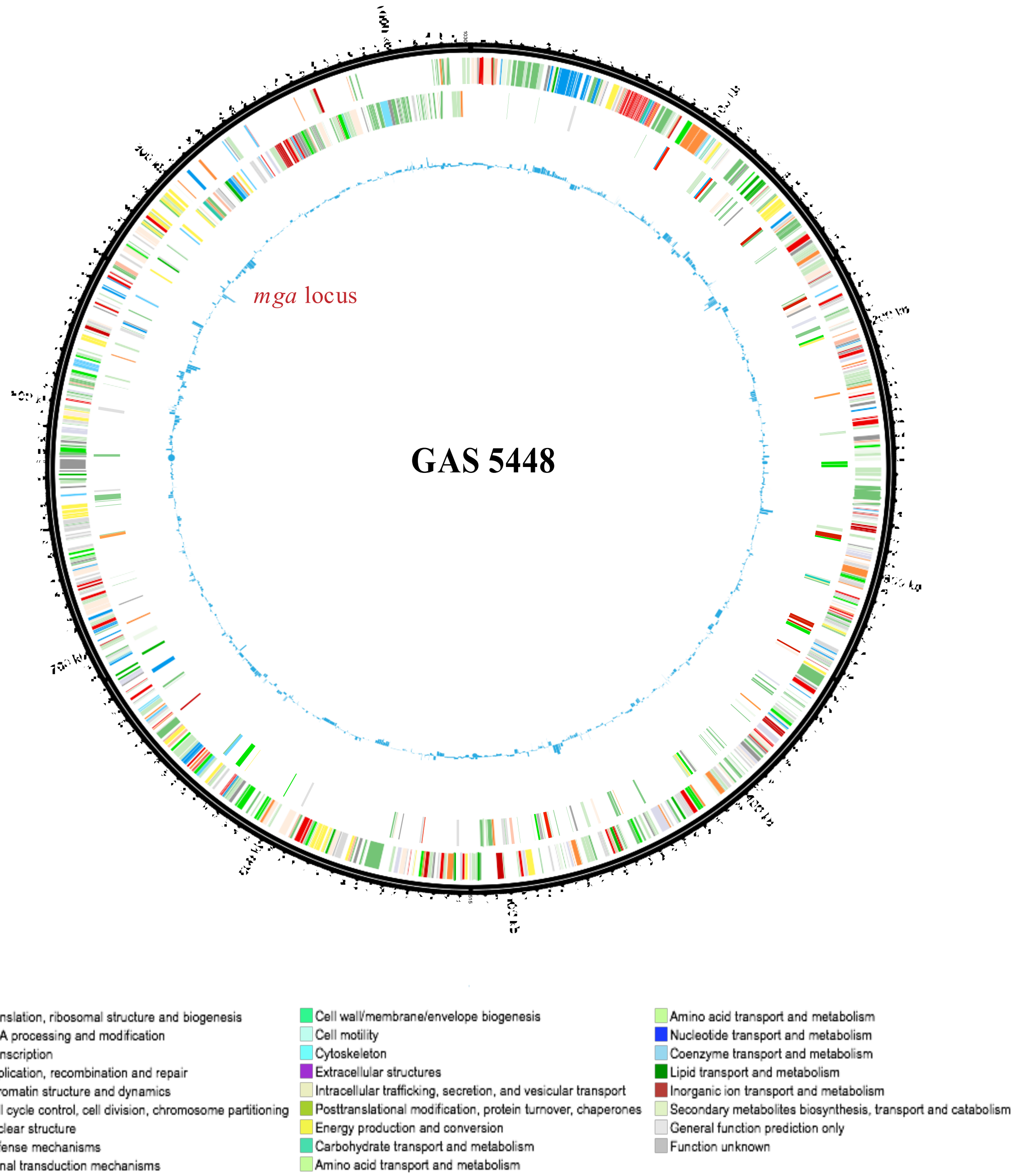

Figure S1. Transcriptomic landscape of M1T1 5448 during growth in C media compared to THY. Circos plot of GAS 5448 differentially expressed (DE) genes during growth in C media (low glucose) versus THY (high glucose) at late logarithmic growth. The outer most ring represents a size ruler and the next two rings represent the GAS open reading frames on the (+) and (-) strand of the genome, respectively, with color representing function COG category as indicted in legend. The inner ring (blue) shows transcript levels of genes, where the height of the bars represent the $\log_2$ fold-change in expression in C media vs. THY (inside is down, outside is up). The position of the *mga* locus is indicated.

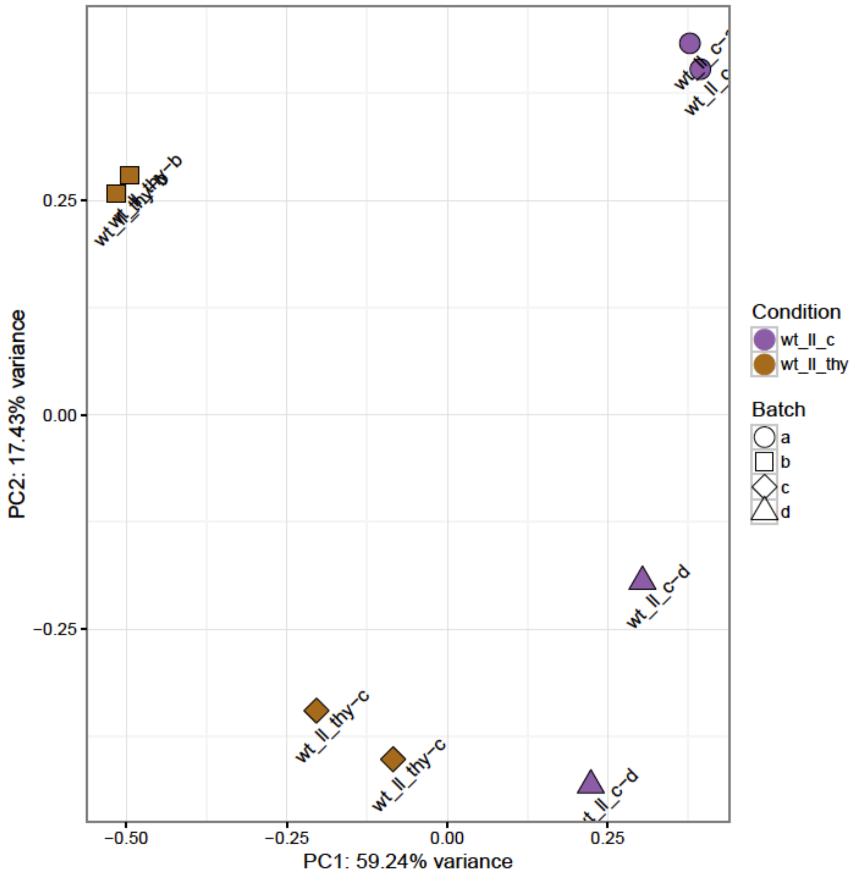

Figure S2. Clustering of WT 5448 samples based on RNA-seq data. A principal component analysis (PCA) plot is shown for the log_2 fold change data of WT 5448 grown in THY (high glucose) or C media (low glucose) at late logarithmic growth phase prior to normalization for batch effects. The PCA plots show the first two principal components on the X and Y axes, respectively, and the percentage of total variance specific to that principle component is indicated. Each biological replicate is represented as a single point with color corresponding to the media condition (THY, brown; C media, purple) and shapes represent different experimental batches.

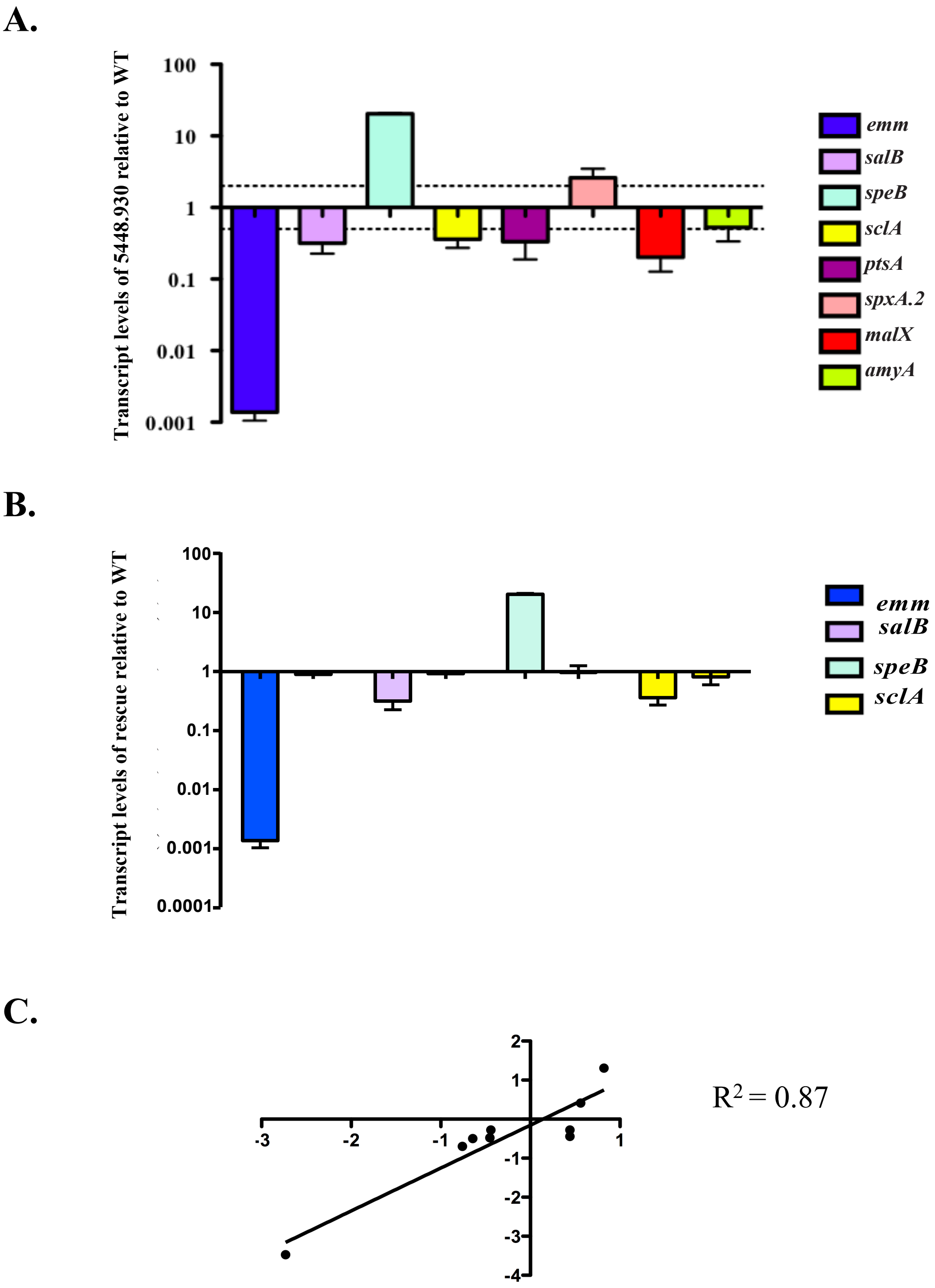

Figure S3. Confirmation of THY RNA-Seq results for Mga regulon using qPCR. (**A**) Transcript levels were determined using qPCR on RNA taken from WT GAS 5448 grown in THY at late-log compared to Δ*mga* 5448.930 grown under the same conditions. Error bars represent the standard error from three biological replicates. Differences greater than 2-fold in expression for WT compared to mutant cells (dashed line) were considered significant. (**B**) Complementation of the Δ*mga* mutant using the rescue strain 5448.930R (hatched bars) restores the transcriptional phenotypes observed in THY. (**C**) Coefficient of correlation between the RNA-Seq and RT-qPCR results generated a confidence in the data with an R^2=87%.

A.

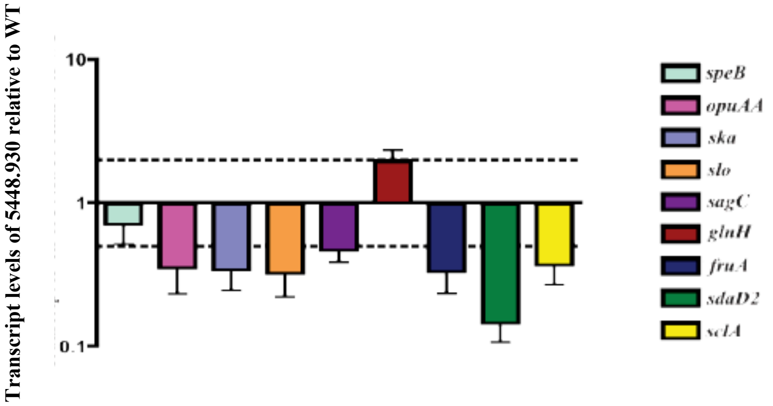

B.

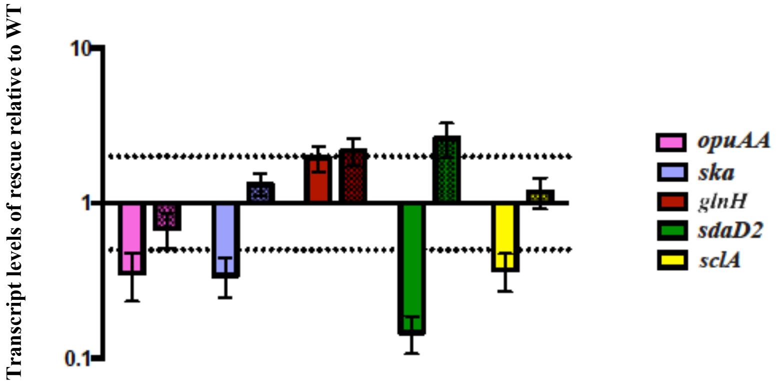

C.

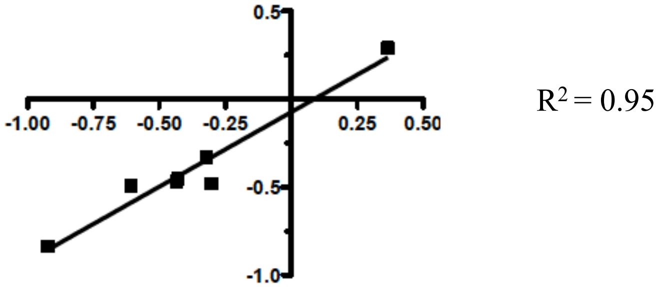

Figure S4. Confirmation of C media RNA-Seq results for Mga regulon using qPCR. (**A**) Transcript levels were determined using qPCR on RNA taken from WT GAS 5448 grown in C media at late-log compared to Δ*mga* 5448.930 grown under the same conditions. Error bars represent the standard error from three biological replicates. Differences greater than 2-fold in expression for WT compared to mutant cells (dashed line) were considered significant. (**B**) Complementation of the Δ*mga* mutant using the rescue strain 5448.930R (hashed bars) restores the transcriptional phenotypes observed in C media. (**C**) Coefficient of correlation between the RNA-Seq and RT-qPCR results. Generated a confidence in the data with an R_2 = 95%.

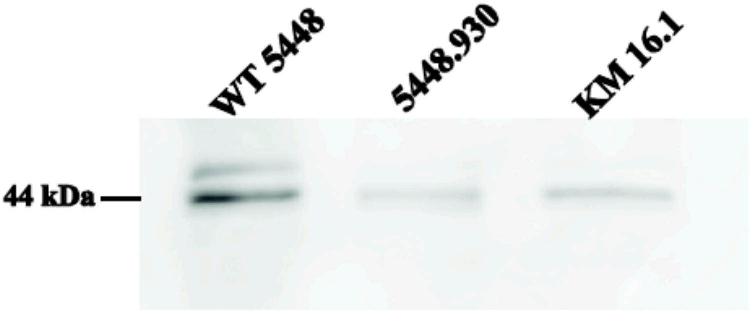

Figure S5. Sda1 levels are reduced in Δ*mga* M1T1 GAS supernatants grown in C media. Western blot analysis of 44-kDa Sda1 streptodornase (encoded by *sdaD2*) in culture supernatants grown to late-log phase (Klett ~70) from WT 5448 and two Δ*mga* mutants (5448.930 and KM16.1). Concentrated culture supernatants were isolated as described in Methods and equal amounts were separated by 10% SDS-PAGE, transferred to nitrocellulose, and probed using a polyclonal α-Sda1 antibody.

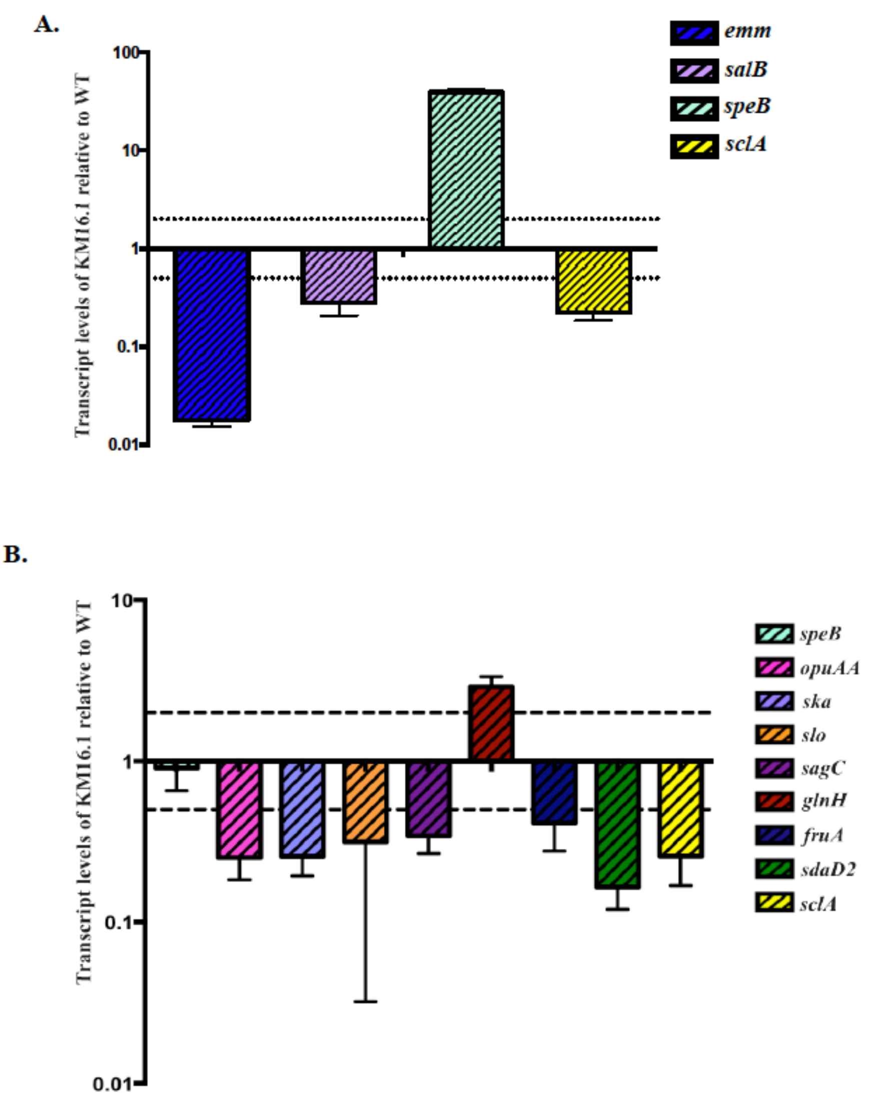

Figure S6. Media-dependent transcriptional regulation by Mga is recapitulated in an independent 5448 Δ*mga* mutant. Transcript levels were determined using qPCR on RNA taken from WT GAS 5448 grown in THY (**A**) or C media (**B**) at late-log compared to Δ*mga* KM16.1 grown under the same conditions. Error bars represent the standard error from three biological replicates. Differences greater than 2-fold in expression for WT compared to mutant cells (dashed line) were considered significant.

Supplemental Table S1: Genes in 5448 differentially expressed (DE) in THY compared to C Media

Spy Number	*cre* (CcpA)[a]	Annotation	Gene	Log$_2$ FC	*p* value
M5005_Spy0011		tRNA(Ile)-lysidine synthetase	*tilS*	-1.45	2.6E-04
M5005_Spy0017	(R[2])	CHAP-domain-containing cell wall hydrolase; pcsB/sibA	*cdhA*	2.34	7.0E-07
M5005_Spy0019	*cre*	DNA repair protein	*recO*	-1.05	1.1E-03
M5005_Spy0022		phosphoribosylaminoimidazole-succinocarboxamide synthase		2.45	1.1E-04
M5005_Spy0023		phosphoribosylformylglycinamidine synthase		1.97	2.7E-04
M5005_Spy0024		amidophosphoribosyltransferase	*purF*	2.76	1.8E-06
M5005_Spy0025		phosphoribosylformylglycinamidine cyclo-ligase	*purM*	2.38	1.0E-04
M5005_Spy0026	*cre*	phosphoribosylaminoimidazolecarboxamide formyltransferase	*purN*	2.05	1.4E-04
M5005_Spy0027		cyclohydrolase		2.00	2.7E-05
M5005_Spy0029		phosphoribosylamine--glycine ligase	*purD*	2.21	7.2E-04
M5005_Spy0030		phosphoribosylaminoimidazole carboxylase catalytic subunit	*purE*	1.63	7.6E-03
M5005_Spy0031		phosphoribosylaminoimidazole carboxylase ATPase subunit	*purK*	1.27	5.1E-03
M5005_Spy0032		hypothetical protein		1.06	9.2E-03
M5005_Spy0034		transcriptional regulator		1.24	3.0E-03
M5005_Spy0035		holliday junction DNA helicase	*ruvB*	1.46	1.5E-04
M5005_Spy0039	*cre2* (R[3,4])	alcohol dehydrogenase/acetaldehyde dehydrogenase	*adh2*	-2.07	6.8E-03
M5005_Spy0040	*cre* (R[2,3])	alcohol dehydrogenase	*adhA*	-1.94	2.3E-02
M5005_Spy0041		Na+ driven multidrug efflux pump		-1.03	9.0E-03
M5005_Spy0043		SSU ribosomal protein S10P	*rpsJ*	1.15	2.2E-02
M5005_Spy0044		LSU ribosomal protein L3P	*rplC*	1.04	1.0E-02
M5005_Spy0052		LSU ribosomal protein L29P	*rpmC*	1.54	1.0E-02
M5005_Spy0064		protein translocase subunit	*secY*	1.22	7.7E-03
M5005_Spy0065		adenylate kinase	*adk*	1.97	4.3E-04
M5005_Spy0066		bacterial protein translation initiation factor 1	*infA*	1.54	3.7E-04
M5005_Spy0068		SSU ribosomal protein S13P	*rpsM*	1.25	1.8E-03
M5005_Spy0070		DNA-directed RNA polymerase alpha chain	*rpoA*	1.49	9.2E-04
M5005_Spy0071		LSU ribosomal protein L17P	*rplQ*	1.70	2.6E-04
M5005_Spy0073		hypothetical protein		2.57	5.5E-03
M5005_Spy0076		4-diphosphocytidyl-2-C-methyl-D-erythritol kinase		1.16	1.7E-02
M5005_Spy0077		transcriptional regulator, MarR family	*adcR*	1.09	8.8E-05
M5005_Spy0078		high-affinity zinc uptake system ATP-binding protein	*adcC*	1.10	1.1E-03
M5005_Spy0085		putative DNA binding protein		2.62	9.1E-06
M5005_Spy0086	*cre*	comG operon protein 1	*comYA*	2.00	5.2E-03
M5005_Spy0088		comG operon protein 3	*comYC*	1.76	2.4E-03
M5005_Spy0094	*cre* (R[2,3])	acetate kinase	*ackA*	1.21	2.0E-03
M5005_Spy0095	(R[4])	hypothetical protein		2.59	1.7E-04
M5005_Spy0106		transcriptional regulator; Regulator of protein F	*rofA*	-1.39	8.0E-04
M5005_Spy0107		fibronectin-binding protein; FCT Pilus Region	*cpa*	2.50	3.8E-07
M5005_Spy0108		signal peptidase I; FCT Pilus Region	*sipA1*	2.52	2.7E-06
M5005_Spy0109		fibronectin-binding protein; FCT Pilus Region		2.37	1.0E-04
M5005_Spy0110		hypothetical protein	*srtC2*	2.61	7.6E-06
M5005_Spy0111		hypothetical protein		1.63	1.6E-03
M5005_Spy0113		transposase		2.14	4.2E-03
M5005_Spy0114		sortase; FCT Pilus Region	*srtB*	2.59	3.0E-04
M5005_Spy0115		hypothetical protein		-1.26	3.9E-04
M5005_Spy0134		tellurite resistance protein		-1.33	1.3E-05
M5005_Spy0137		nucleoside-binding protein		1.39	1.6E-05
M5005_Spy0142		hypothetical protein		2.34	2.5E-03
M5005_Spy0143	*cre*	hypothetical protein		3.97	1.9E-03
M5005_Spy0148	*cre*	PTS system, **3-keto-L-gulonate** specific IIC	*ptxC*	-2.37	5.9E-03
M5005_Spy0149	(R[4])	PTS system, **3-keto-L-gulonate** specific IIB	*ptxB*	-4.53	2.0E-05
M5005_Spy0150	(R[4])	PTS system, **3-keto-L-gulonate** specific IIA	*ptxA*	-4.61	3.3E-04
M5005_Spy0151	(R[2,4])	3-keto-L-gulonate-6-phosphate decarboxylase		-3.52	4.3E-03
M5005_Spy0152		L-xylulose 5-phosphate 3-epimerase		-3.57	7.4E-04
M5005_Spy0153		L-ribulose-5-phosphate 4-epimerase	*araD*	-5.77	3.5E-06
M5005_Spy0154		hypothetical protein		-3.95	5.7E-05
M5005_Spy0156		metal-dependent hydrolase		-2.14	4.0E-04
M5005_Spy0157	(A[3])	glycine betaine transport ATP-binding protein	*opuAA*	2.11	1.7E-07
M5005_Spy0158		glycine betaine transport system permease protein	*opuABC*	2.01	2.5E-06
M5005_Spy0174		hypothetical protein		1.96	1.2E-02
M5005_Spy0175		queuine tRNA-ribosyltransferase	*tgt*	1.06	2.1E-03
M5005_Spy0177		bioY protein		1.21	2.0E-02
M5005_Spy0185	*cre*	glucose-6-phosphate isomerase	*pgi*	1.09	3.8E-02
M5005_Spy0192		UTP--glucose-1-phosphate uridylyltransferase	*hasC.2*	-1.19	1.4E-03

M5005_Spy0194	(R[4])	glycerol-3-phosphate dehydrogenase; GAPDH	*gapA*	-1.27	2.1E-04
M5005_Spy0195		Heme-sensitive transcriptional repressor, MarR-family	*pefR*	1.17	1.3E-02
M5005_Spy0204		TCS-1 histidine kinase FasB	*fasB*	1.10	1.4E-03
M5005_Spy0206		TCS-1 response regulator FasA	*fasA*	1.13	1.0E-02
M5005_Spy0211		LSU ribosomal protein L34P	*rpmH*	1.64	1.6E-03
M5005_Spy0213	(R[2,3,4])	ABC **Sialic Acid**; N-acetylneuraminate-binding protein		-1.98	1.6E-03
M5005_Spy0214	*cre* (R[2,3,4])	ABC **Sialic Acid**; N-acetylneuraminate permease protein		-2.11	5.6E-04
M5005_Spy0215	(R[2,3,4])	ABC **Sialic Acid**; N-acetylneuraminate permease protein		-1.83	3.2E-03
M5005_Spy0216	(R[2,3,4])	hypothetical membrane spanning protein		-2.87	1.1E-04
M5005_Spy0217	(R[2,3,4])	N-acetylneuraminate lyase	*nanH*	-1.94	3.3E-03
M5005_Spy0218	(R[3,4])	N-acetylmannosamine kinase		-1.95	2.8E-03
M5005_Spy0221		ribonuclease M5		1.31	1.8E-03
M5005_Spy0224		ribulose-phosphate 3-epimerase	*rpe*	-1.36	1.2E-04
M5005_Spy0230		SSU ribosomal protein S12P	*rpsL*	1.71	4.2E-04
M5005_Spy0231		SSU ribosomal protein S7P	*rpsG*	1.32	4.4E-03
M5005_Spy0238		putative undecaprenol kinase	*bacA*	-1.20	1.7E-03
M5005_Spy0241		hypothetical cytosolic protein	*rgpG*	1.62	2.5E-02
M5005_Spy0247		D-alanyl-D-alanine carboxypeptidase		1.79	1.2E-05
M5005_Spy0273		ABC transporter permease protein		1.32	4.4E-02
M5005_Spy0274	*cre*	branched-chain amino acid transport system carrier protein	*braB*	4.77	5.8E-07
M5005_Spy0275	*cre*	serine/threonine sodium symporter		3.26	2.2E-07
M5005_Spy0295		60 kDa inner membrane protein YIDC		-1.20	1.6E-03
M5005_Spy0296		acylphosphatase		-1.23	2.0E-03
M5005_Spy0307		DNA integration/recombination/invertion protein		-1.22	1.3E-04
M5005_Spy0318		pyruvate formate-lyase activating enzyme	*pflC*	1.35	3.5E-04
M5005_Spy0321	(R[2])	ferrichrome transport system permease protein	*fhuG*	1.34	5.2E-04
M5005_Spy0323	(R[2])	ferrichrome-binding protein	*fhuD*	1.13	7.5E-03
M5005_Spy0324	(R[2])	ferrichrome transport ATP-binding protein	*fhuA*	1.85	1.2E-04
M5005_Spy0329		hypothetical cytosolic protein		-1.11	5.1E-05
M5005_Spy0333		signal peptidase-like protein		-1.23	4.7E-04
M5005_Spy0335		corrin/porphyrin methyltransferase		-0.97	1.8E-04
M5005_Spy0336		hypothetical membrane associated protein		-1.13	1.0E-02
M5005_Spy0338		arsenate reductase family protein		1.24	1.3E-03
M5005_Spy0341	*cre*	lactocepin; SpyCEP; ScpC; IL-8 protease	*spyCEP*	-1.78	1.4E-04
M5005_Spy0346	(R[2])	hypothetical protein		-1.41	2.8E-02
M5005_Spy0347	(R[2])	ribonucleoside-diphosphate reductase beta chain	*nrdF.1*	-1.25	2.0E-03
M5005_Spy0348	(R[2])	hypothetical protein	*nrdI*	-1.49	5.0E-03
M5005_Spy0349	(R[2])	ribonucleoside-diphosphate reductase alpha chain	*nrdE.1*	-1.56	4.3E-03
M5005_Spy0361	*cre* (R[2,4])	phosphoglycerate transporter protein		-1.05	3.3E-02
M5005_Spy0374		LSU ribosomal protein L11P	*rplK*	1.84	4.9E-03
M5005_Spy0375		LSU ribosomal protein L1P	*rplA*	1.86	3.2E-03
M5005_Spy0385	*cre*	67 kDa Myosin-crossreactive antigen		-1.23	5.2E-03
M5005_Spy0387		uracil DNA glycosylase superfamily protein		-0.98	1.3E-02
M5005_Spy0398		bacteriocin		1.53	4.0E-02
M5005_Spy0421		lactoylglutathione lyase	*gloA*	-1.70	1.2E-05
M5005_Spy0422		NAD(P)H-dependent quinone reductase		-1.94	4.0E-06
M5005_Spy0423	*cre2*	Xaa-Pro dipeptidase	*pepQ*	-1.61	1.8E-04
M5005_Spy0425		glycosyltransferase		-1.29	2.1E-04
M5005_Spy0426		1,2-diacylglycerol 3-glucosyltransferase		-1.52	2.8E-04
M5005_Spy0457		plasmid stabilization system protein		-1.08	2.5E-02
M5005_Spy0461		hypothetical cytosolic protein		-1.67	1.6E-04
M5005_Spy0462		hypothetical protein		-1.50	6.6E-03
M5005_Spy0463		hypothetical cytosolic protein		-1.13	5.4E-03
M5005_Spy0464		microcin C7 self-immunity protein	*mccF*	-1.14	1.3E-03
M5005_Spy0465		hypothetical protein		-0.95	1.5E-02
M5005_Spy0466		hypothetical protein		-1.43	2.9E-05
M5005_Spy0467		transposase		-1.09	2.8E-02
M5005_Spy0469		hypothetical protein		-1.74	9.1E-04
M5005_Spy0473		multidrug resistance protein B		1.46	1.4E-03
M5005_Spy0474	(R[2,3,4])	transcription antiterminator, BglG family	*licT*	2.43	7.5E-05
M5005_Spy0475	*cre* (R[2,3,4])	PTS system, **b-Glucoside**-specific IIABC		3.78	2.7E-08
M5005_Spy0476	(R[2,3,4])	6-phospho-beta-glucosidase	*bglA*	2.94	4.4E-06
M5005_Spy0477		hypothetical membrane spanning protein		-1.67	3.6E-05
M5005_Spy0478		hypothetical membrane spanning protein		-1.65	3.7E-04
M5005_Spy0479		hypothetical membrane spanning protein		-3.11	5.0E-02
M5005_Spy0483		stress-responsive transcriptional regulator		-2.31	4.4E-08
M5005_Spy0485		prolipoprotein diacylglyceryl transferase	*lgt*	-0.96	5.6E-04
M5005_Spy0486		hypothetical protein		-1.21	3.0E-03
M5005_Spy0496		hydrolase, HAD superfamily		1.90	5.5E-06

M5005_Spy0503		glutathione peroxidase		-1.45	1.2E-04
M5005_Spy0504		oligoendopeptidase F	*pepF*	-1.70	1.0E-05
M5005_Spy0512	*cre*	hydrolase, HAD superfamily		-1.02	8.9E-05
M5005_Spy0523		hypothetical protein		-1.63	2.3E-02
M5005_Spy0528		glucose-1-phosphate phosphodismutase		-1.13	1.2E-03
M5005_Spy0539		ATP-binding protein		-1.30	2.2E-05
M5005_Spy0540		transporter		-1.27	4.1E-04
M5005_Spy0541		hypothetical cytosolic protein		-1.50	6.2E-05
M5005_Spy0542		dipeptidase	*pepD*	-1.77	8.8E-05
M5005_Spy0543	*cre2*	high-affinity zinc uptake system protein znuA precursor		1.49	3.5E-03
M5005_Spy0545		galactosamine-6-phosphate deaminase (isomerizing)	*agaS*	-0.93	1.5E-02
M5005_Spy0546		LSU ribosomal protein L31P	*rpmE*	1.77	5.0E-06
M5005_Spy0549	*cre*	chorismate mutase		1.13	1.0E-02
M5005_Spy0551		LSU ribosomal protein L19P	*rplS*	2.14	4.6E-05
M5005_Spy0555		hypothetical cytosolic protein		-1.40	7.8E-04
M5005_Spy0558		transposase		-1.01	4.6E-02
M5005_Spy0562	(R[2,3,4])	streptolysin S precursor	*sagA*	-2.37	2.1E-04
M5005_Spy0563	(R[2,3,4])	streptolysin S biosynthesis protein	*sagB*	-1.86	1.9E-04
M5005_Spy0564	(R[2,3,4])	streptolysin S biosynthesis protein	*sagC*	-1.87	1.0E-04
M5005_Spy0565	(R[2,3,4])	streptolysin S biosynthesis protein	*sagD*	-1.25	1.9E-03
M5005_Spy0566	(R[2,3,4])	streptolysin S putative self-immunity protein	*sagE*	-2.32	5.5E-05
M5005_Spy0567	(R[2,3,4])	streptolysin S biosynthesis protein	*sagF*	-1.82	1.0E-03
M5005_Spy0568	(R[2,3,4])	streptolysin S export ATP-binding protein	*sagG*	-1.88	7.7E-05
M5005_Spy0569	(R[2,3,4])	streptolysin S export transmembrane protein	*sagH*	-1.76	1.9E-04
M5005_Spy0570	(R[2,3,4])	streptolysin S export transmembrane protein	*sagI*	-1.14	2.7E-02
M5005_Spy0575		ATP synthase C chain	*atpE*	1.60	1.4E-04
M5005_Spy0576	(A[3])	ATP synthase A chain	*atpB*	1.26	1.7E-03
M5005_Spy0579	(A[3])	ATP synthase alpha chain	*atpA*	1.43	2.0E-02
M5005_Spy0593		neutral zinc metallopeptidase family		-1.35	7.7E-05
M5005_Spy0594		ATP-dependent nuclease subunit B	*rexB*	-1.18	6.8E-04
M5005_Spy0595		ATP-dependent nuclease subunit A	*rexA*	-1.26	4.1E-04
M5005_Spy0597		SSU ribosomal protein S21P		1.39	1.1E-03
M5005_Spy0611		Group A Carbohydrate; Membrane protein	*gacJ*	-1.02	8.6E-03
M5005_Spy0615		pore forming protein	*ebsA*	-1.66	3.5E-04
M5005_Spy0616		ferredoxin		-1.78	4.7E-02
M5005_Spy0618		cytidylate kinase	*cmk*	-1.08	4.3E-04
M5005_Spy0619		bacterial protein translation initiation factor 3	*infC*	2.03	4.4E-04
M5005_Spy0620	*cre*	LSU ribosomal protein L35P	*rpl36*	1.93	2.3E-03
M5005_Spy0621		LSU ribosomal protein L20P	*rplT*	2.34	1.2E-05
M5005_Spy0624		3-dehydroquinate dehydratase	*aroD*	1.25	4.3E-04
M5005_Spy0625		chorismate synthase	*aroF*	1.20	5.5E-03
M5005_Spy0628		folylpolyglutamate synthase/dihydrofolate synthase	*folC.2*	-1.18	1.5E-04
M5005_Spy0633		LSU ribosomal protein L21P	*rplU*	1.84	3.9E-05
M5005_Spy0634		hypothetical ribosome-associated protein		1.05	1.4E-03
M5005_Spy0635		LSU ribosomal protein L27P	*rpmA*	2.05	2.7E-05
M5005_Spy0636		transcriptional regulator, LysR family		-1.36	2.0E-05
M5005_Spy0637		lipoprotein signal peptidase	*lsp*	-1.45	5.0E-05
M5005_Spy0638		ribosomal large subunit pseudouridine synthase D		-1.21	1.1E-05
M5005_Spy0639		pyrR bifunctional protein	*pyrR*	1.57	1.6E-03
M5005_Spy0640		uracil permease	*pyrP*	1.30	5.0E-03
M5005_Spy0641		aspartate carbamoyltransferase	*pyrB*	2.18	1.7E-05
M5005_Spy0642		carbamoyl-phosphate synthase small chain	*carA*	1.73	3.8E-04
M5005_Spy0643		carbamoyl-phosphate synthase large chain	*carB*	1.62	1.6E-03
M5005_Spy0648		SSU ribosomal protein S16P	*rpsP*	1.74	4.3E-04
M5005_Spy0649		RNA binding protein		1.59	7.4E-05
M5005_Spy0651	(R[2])	Heme-utilization cell surface protein	*hupY*	1.11	1.0E-02
M5005_Spy0652		Heme-oxygenase cytosolic protein	*hupZ*	1.76	4.9E-04
M5005_Spy0653	*cre*	cobalt-zinc-cadmium resistance protein	*czcD*	-3.16	3.4E-05
M5005_Spy0655		16S rRNA processing protein	*rimM*	1.25	6.3E-04
M5005_Spy0668		IgG-degrading protease	*mac*	1.82	4.0E-03
M5005_Spy0669		phage protein		1.84	3.4E-02
M5005_Spy0676		hypothetical protein		1.25	1.6E-02
M5005_Spy0686		3-hydroxy-3-methylglutaryl-coenzyme A reductase		-1.29	5.4E-04
M5005_Spy0687		hydroxymethylglutaryl-CoA synthase	*mvaS.1*	-0.95	1.5E-02
M5005_Spy0696	(R[4])	phosphopentomutase	*deoB*	-1.00	7.2E-03
M5005_Spy0698	(R[4])	purine nucleoside phosphorylase	*punA*	-1.01	2.4E-02
M5005_Spy0703		orotidine 5'-phosphate decarboxylase	*pyrF*	1.62	6.4E-03
M5005_Spy0704		orotate phosphoribosyltransferase	*pyrE*	2.61	1.4E-03
M5005_Spy0706		cystine-binding protein		1.67	4.8E-05

M5005_Spy0707		cystine transport system permease protein		1.07	8.2E-03
M5005_Spy0717	*cre*	rhodanese-related sulfurtransferases		1.23	6.7E-04
M5005_Spy0718		hypothetical protein		1.11	7.4E-03
M5005_Spy0719		glutathione S-transferase		2.40	2.2E-06
M5005_Spy0721		hypothetical protein		-0.99	3.2E-02
M5005_Spy0732		NIF3-related protein		1.24	2.4E-02
M5005_Spy0735		dTDP-4-dehydrorhamnose 3,5-epimerase	*cpsFP*	-1.04	8.1E-04
M5005_Spy0737		mutator mutT protein/7,8-dihydro-8-oxoguanine-triphosphatase		-1.48	6.3E-05
M5005_Spy0738		hypothetical membrane spanning protein		-1.64	1.7E-06
M5005_Spy0739		tetratricopeptide repeat family protein		-1.19	1.2E-03
M5005_Spy0751		pyruvate dehydrogenase E1 component alpha subunit	*acoA*	1.95	5.3E-03
M5005_Spy0752		pyruvate dehydrogenase E1 component beta subunit	*acoB*	1.85	9.3E-04
M5005_Spy0753		dihydrolipoamide acetyltransferase of pyruvate dehydrogenase	*acoC*	1.02	5.2E-03
M5005_Spy0774		nucleoside diphosphate kinase		2.02	5.4E-03
M5005_Spy0775		nucleoside diphosphate kinase		1.61	6.3E-04
M5005_Spy0780	*cre* (R[2,3,4])	PTS system, **Mannose/Fructose** family IIA component	*ptsA*	1.51	7.6E-03
M5005_Spy0781	(R[2,3,4])	PTS system, **Mannose/Fructose** family IIB component	*ptsB*	1.33	1.3E-03
M5005_Spy0783	(R[2,3,4])	PTS system, **Mannose/Fructose** family IID component	*ptsD*	1.12	1.0E-02
M5005_Spy0790	*cre* (R[3,4])	succinate-semialdehyde dehydrogenase	*gabD*	-1.23	1.5E-02
M5005_Spy0791	(R[4])	excinuclease ABC subunit C	*uvrC*	-1.98	5.5E-04
M5005_Spy0792		NAD(P)H-dependent quinone reductase		-2.64	1.2E-04
M5005_Spy0793		Xaa-His dipeptidase		-1.74	1.8E-02
M5005_Spy0794		tRNA (5-carboxymethylaminomethyl-2-thiouridylate) synthase	*thdF*	2.16	4.5E-06
M5005_Spy0795		LSU ribosomal protein L10P	*rplJ*	1.52	9.0E-03
M5005_Spy0796		LSU ribosomal protein L12P	*rplL*	2.69	1.0E-03
M5005_Spy0804		TCS-6 response regulator; Streptin biosynthesis	*srtR*	-0.94	3.6E-04
M5005_Spy0806		lantibiotic precursor	*srtA*	-1.24	2.1E-03
M5005_Spy0807		ABC transporter (ATP binding)-lantibiotic associated	*srtT*	-1.63	6.4E-06
M5005_Spy0808		lantibiotic transport ATP-binding protein	*srtF*	-0.95	3.5E-03
M5005_Spy0809		lantibiotic transport permease protein	*srtE*	-1.98	3.8E-05
M5005_Spy0810		lantibiotic transport permease protein	*srtG*	-1.62	1.1E-04
M5005_Spy0811		transcriptional regulator, Cro/CI family		-1.29	4.6E-02
M5005_Spy0820		tetrahydropholate synth./dihydrofolate synthase	*folC.1*	-2.04	5.6E-08
M5005_Spy0821	*cre2*	tetrahydropholate synth., GTP cyclohydrolase I	*folE*	-1.20	3.7E-04
M5005_Spy0822		tetrahydropholate synth., dihydropteroate synthase	*folP*	-1.11	2.1E-02
M5005_Spy0823		tetrahydropholate synth., dihydroneopterin aldolase	*folQ*	-1.34	3.4E-04
M5005_Spy0824		tetrahydropholate synth., pyrophosphokinase	*folK*	-1.51	1.2E-04
M5005_Spy0825		UDP-N-acetylenolpyruvoylglucosamine reductase	*murB*	1.29	2.1E-04
M5005_Spy0826		spermidine/putrescine transport system ATP-binding protein	*potA*	2.58	4.0E-05
M5005_Spy0827		spermidine/putrescine transport system permease protein	*potB*	1.36	7.8E-04
M5005_Spy0828		spermidine/putrescine transport system permease protein	*potC*	1.52	4.0E-03
M5005_Spy0829		spermidine/putrescine-binding protein		2.29	3.4E-04
M5005_Spy0835	*cre* (R[2,3])	class B acid phosphatase		-1.26	2.4E-03
M5005_Spy0841		glutamine amidotransferase, class I		-1.06	2.8 E-03
M5005_Spy0843		hypothetical cytosolic protein		-1.46	7.7E-05
M5005_Spy0845		ribose-phosphate pyrophosphokinase		-1.00	1.7E-03
M5005_Spy0852	(R[3,4])	short chain dehydrogenase		2.02	1.6E-06
M5005_Spy0853	(R[3])	short chain dehydrogenase		2.86	4.2E-07
M5005_Spy0858	(A[4])	xanthine phosphoribosyltransferase	*xpt*	4.52	1.5E-06
M5005_Spy0859	(A[4])	xanthine permease		4.41	1.6E-09
M5005_Spy0873	*cre*	L-lactate dehydrogenase	*ldh*	1.60	1.4E-03
M5005_Spy0879		hypothetical protein		-2.03	3.5E-04
M5005_Spy0880		conserved membrane protein		-5.13	1.1E-06
M5005_Spy0881	*cre*	hypothetical cytosolic protein		-5.63	7.1E-09
M5005_Spy0883		ribonuclease HII		1.06	3.5 E-05
M5005_Spy0884		hypothetical protein	*smf*	2.51	1.1E-08
M5005_Spy0890		D-lactate dehydrogenase	*ddh*	1.57	6.8E-03
M5005_Spy0911		hypothetical protein		-1.01	8.4E-03
M5005_Spy0914		phage transcriptional repressor		1.30	5.0E-03
M5005_Spy0943	*cre*	cytidine deaminase	*cdd*	1.42	5.4E-04
M5005_Spy0945	*cre* (R[4])	pantothenate kinase	*coaA*	-1.61	4.4E-04
M5005_Spy0965		ABC transporter permease protein		-1.09	6.2E-04
M5005_Spy0982	*cre* (R[2],A[4])	histidine-binding protein		1.14	1.3E-03
M5005_Spy1017		phage protein		3.15	6.5E-03
M5005_Spy1023		terminase large subunit		1.45	3.6E-02
M5005_Spy1028		phage protein		3.67	1.9E-03
M5005_Spy1046		phage protein		-1.28	5.0E-03
M5005_Spy1062		maltodextrose utilization protein	*malA*	-1.96	4.0E-02
M5005_Spy1063	(R[2,3])	ABC **Cyclomaltodextrin** permease protein	*malD*	-1.85	3.9E-02

M5005_Spy1064	($R^{2,3}$)	ABC **Cyclomaltodextrin** permease protein	*malC*	-1.57	5.1E-02
M5005_Spy1065	($R^{2,3}$)	alpha-amylase	*amyA*	-1.70	2.9E-02
M5005_Spy1066	($R^{2,3}$)	neopullulanase/cyclomaltodextrinase/maltogenic alpha-amylase	*amyB*	-1.65	5.4E-02
M5005_Spy1069	($R^{2,3}$)	esterase		-0.99	2.8E-03
M5005_Spy1070		protein precursor	*dltD*	-0.92	1.0E-02
M5005_Spy1072		protein DltB	*dltB*	-1.47	6.7E-05
M5005_Spy1073		D-alanine-activating enzyme	*dltA*	-1.43	1.0E-06
M5005_Spy1074		hypothetical protein		-1.79	3.1E-04
M5005_Spy1075		excinuclease ABC subunit B	*uvrB*	-1.57	6.0E-07
M5005_Spy1079	($R^{2,4}$)	PTS system, **Cellobiose**-specific IIC component		-2.09	3.4E-03
M5005_Spy1080	($R^{2,4}$)	hypothetical protein		-1.43	6.7E-03
M5005_Spy1081	($R^{2,4}$)	PTS system, **Cellobiose**-specific IIA component		-1.76	8.4E-03
M5005_Spy1082	($R^{2,4}$)	PTS system, **Cellobiose**-specific IIB component		-1.99	4.1E-03
M5005_Spy1083	*cre* ($R^{2,4}$)	PTS system, **Cellobiose**-specific IIA component		-1.83	5.8E-04
M5005_Spy1084	($R^{2,4}$)	outer surface protein		-1.25	2.2E-02
M5005_Spy1085	*cre* (R^{4})	beta-glucosidase	*bglA.2*	-1.61	6.6E-03
M5005_Spy1093	*cre* ($R^{2,3,4}$)	hypothetical protein		-3.31	2.3E-06
M5005_Spy1096		thioesterase superfamily protein		1.85	7.6E-03
M5005_Spy1097		phosphorylase, Pnp/Udp family		-0.97	2.2E-02
M5005_Spy1100		shikimate kinase	*aroK*	-1.23	4.6E-03
M5005_Spy1102		ribonuclease BN		-1.46	1.7E-04
M5005_Spy1106	*cre*	protein G-related alpha 2M-binding protein	*grab*	1.90	7.1E-05
M5005_Spy1107		UDP-N-acetylglucosamine 1-carboxyvinyl transferase	*murZ*	1.34	6.5E-03
M5005_Spy1109		internalin protein	*inlA*	1.27	1.0E-03
M5005_Spy1114		hypothetical membrane spanning protein		-1.93	9.0E-04
M5005_Spy1117		ATP-dependent RNA helicase	*deaD2*	-1.00	4.3E-03
M5005_Spy1120		phosphoenolpyruvate-protein phosphotransferase	*pstI*	1.55	6.2E-04
M5005_Spy1122		glutaredoxin	*nrdH*	-1.87	4.9E-05
M5005_Spy1123		ribonucleoside-diphosphate reductase alpha chain	*nrdE.2*	-1.62	1.0E-03
M5005_Spy1124		ribonucleoside-diphosphate reductase beta chain	*nrdF.2*	-1.07	4.8E-03
M5005_Spy1125		chloride channel protein		-1.59	3.1E-06
M5005_Spy1131		transcriptional regulator, Cro/CI family		2.11	3.7E-05
M5005_Spy1132		alanyl-tRNA synthetase	*alaS*	2.46	9.0E-06
M5005_Spy1137		putative competence protein/transcription factor		1.62	2.3E-03
M5005_Spy1139		glucosamine-6-phosphate isomerase	*nagB*	1.24	2.7E-03
M5005_Spy1142		hypothetical protein		-2.17	9.2E-03
M5005_Spy1143		hypothetical protein		-2.16	1.9E-02
M5005_Spy1144	*cre*	hypothetical protein		-2.26	1.1E-03
M5005_Spy1149		1-acyl-sn-glycerol-3-phosphate acyltransferase		1.23	3.3E-02
M5005_Spy1152		kup system potassium uptake protein		1.56	3.1E-04
M5005_Spy1167		Pb, Cd, Zn and Hg-transporting ATPase	*pmtA*	1.43	1.9E-03
M5005_Spy1199		phage protein		2.11	2.8E-02
M5005_Spy1205		phage protein		1.51	1.0E-02
M5005_Spy1227		hypothetical protein		1.08	2.6E-03
M5005_Spy1229		arginine repressor	*argR1*	1.72	7.1E-03
M5005_Spy1230		hemolysin		1.18	5.0E-02
M5005_Spy1231		dimethylallyltransferase/geranyltranstransferase	*fps*	1.55	1.7E-02
M5005_Spy1232		exodeoxyribonuclease VII small subunit	*xseB*	2.43	1.1E-03
M5005_Spy1237	(R^{2})	arginine transport ATP-binding protein	*artP*	2.19	7.8E-05
M5005_Spy1238	(R^{2})	arginine transport system permease protein	*artQ*	1.52	1.3E-03
M5005_Spy1241		mutator protein (7,8-dihydro-8-oxoguanine-triphosphatase)	*mutT*	-0.95	2.1E-03
M5005_Spy1256	(R^{4})	rhodanese-related sulfurtransferases		1.12	8.3E-03
M5005_Spy1257	(R^{3})	glucokinase/Xylose repressor	*glcK*	1.98	2.7E-04
M5005_Spy1258	*cre*	hypothetical cytosolic protein		2.50	3.0E-06
M5005_Spy1263		hypothetical protein		-1.65	3.3E-03
M5005_Spy1270	($R^{2,3,4}$)	carbamate kinase	*arcC*	-3.88	3.1E-05
M5005_Spy1271	($R^{2,3,4}$)	Xaa-His dipeptidase		-4.11	5.7E-09
M5005_Spy1272	($R^{2,3,4}$)	arginine/ornithine antiporter		-4.09	2.0E-07
M5005_Spy1273	($R^{2,3,4}$)	ornithine carbamoyltransferase	*arcB*	-4.04	3.8E-06
M5005_Spy1274	($R^{2,3,4}$)	acetyltransferase		-3.79	1.9E-07
M5005_Spy1275	*cre* ($R^{2,3,4}$)	arginine deiminase	*arcA*	-3.22	2.5E-04
M5005_Spy1281		TCS-9 response regulator yesN-like	*tcs9R*	-0.99	1.6E-02
M5005_Spy1297		phospho-2-dehydro-3-deoxyheptonate aldolase		1.91	7.1E-04
M5005_Spy1298		3-dehydroquinate synthase	*aroB*	2.02	8.4E-03
M5005_Spy1299		hypothetical protein		-1.91	4.4E-02
M5005_Spy1305		TCS-10 response regulator; TCS-X; YesN-like	*trxR*	-1.20	1.4E-02
M5005_Spy1308		ABC unknown sugar-binding protein		-1.79	8.8E-03
M5005_Spy1309		ABC unknown sugar permease protein		-2.78	7.0E-03
M5005_Spy1310	*cre2*	ABC unknown sugar permease protein		-2.82	6.2E-03

M5005_Spy1311	*cre*	glucokinase		-1.95	2.4E-03
M5005_Spy1312		hypothetical protein		-3.23	3.4E-03
M5005_Spy1319		tRNA (uracil-5-)-methyltransferase		1.24	2.0E-02
M5005_Spy1323		transposase		-1.12	2.0E-02
M5005_Spy1329	(A[3])	cysteine synthase	*cysM*	3.54	1.0E-05
M5005_Spy1334		transporter	*yvqF*	-1.13	1.0E-02
M5005_Spy1340		DNA-directed RNA polymerase omega chain		2.04	1.9E-05
M5005_Spy1341		guanylate kinase	*gmk*	1.23	4.2E-03
M5005_Spy1342		hydrolase, HAD superfamily		1.30	4.2E-04
M5005_Spy1344		acetyl-CoA acetyltransferase	*atoB*	-1.16	5.2E-04
M5005_Spy1346		acetate CoA-transferase beta subunit	*atoA*	-1.44	6.9E-04
M5005_Spy1347		D-beta-hydroxybutyrate dehydrogenase		-1.76	1.0E-03
M5005_Spy1348		D-beta-hydroxybutyrate permease		-2.18	5.5E-08
M5005_Spy1354		recombination protein	*recU*	-1.14	3.5E-03
M5005_Spy1356	*cre*	aminopeptidase C	*pepC*	-1.25	1.8E-02
M5005_Spy1359		amino acid permease		1.36	1.8E-04
M5005_Spy1362		transporter		1.78	4.0E-06
M5005_Spy1363		amino acid ABC transporter permease protein		1.17	1.6E-03
M5005_Spy1374		hypothetical protein		1.17	1.4E-02
M5005_Spy1376	*cre* (R[2,3,4])	transaldolase		-2.82	1.0E-04
M5005_Spy1377	(R[2,3,4])	trans-acting positive regulator		-2.26	3.4E-03
M5005_Spy1378	*cre* (R[2,3,4])	NADH peroxidase		-1.90	1.7E-02
M5005_Spy1379	(R[2,3,4])	glycerol uptake facilitator protein	*glpF*	-4.84	1.0E-05
M5005_Spy1380	(R[2,4])	alpha-glycerophosphate oxidase	*glpO*	-4.44	2.1E-07
M5005_Spy1381	*cre* (R[2,3,4])	glycerol kinase	*glpK*	-4.13	5.4E-08
M5005_Spy1391		degV family protein		-1.76	7.6E-06
M5005_Spy1393		hydrolase, HAD superfamily		-1.17	5.1E-05
M5005_Spy1395	(R[2,4])	tagatose-bisphosphate aldolase; SpeB regulator	*lacD.1*	-2.93	2.7E-04
M5005_Spy1396	(R[2])	tagatose-6-phosphate kinase	*lacC.1*	-2.29	6.0E-03
M5005_Spy1397	(R[2,4])	galactose-6-phosphate isomerase lacB subunit	*lacB.1*	-2.54	8.1E-04
M5005_Spy1398	(R[2,4])	galactose-6-phosphate isomerase lacA subunit	*lacA.1*	-2.63	4.2E-03
M5005_Spy1399	(R[2,4])	PTS system, **Galactose**-specific IIC component		-2.16	2.1E-02
M5005_Spy1408		ribosome-binding factor A	*rbfA*	1.44	2.4E-03
M5005_Spy1409		bacterial protein translation initiation factor 2	*infB*	1.69	2.0E-04
M5005_Spy1410		LSU ribosomal protein L7AE		1.47	2.8E-04
M5005_Spy1412		N utilization substance protein A	*nusA*	1.09	4.2E-03
M5005_Spy1420		phage protein		1.52	3.1E-03
M5005_Spy1428		phage protein		1.79	3.1E-03
M5005_Spy1432		phage protein		1.08	3.2E-02
M5005_Spy1441		phage terminase small subunit		1.52	1.3E-02
M5005_Spy1442		phage transcriptional activator		2.16	2.7E-04
M5005_Spy1444		adenine-specific methyltransferase		2.09	5.8E-03
M5005_Spy1445		phage protein		2.05	5.4E-03
M5005_Spy1447		phage-related DNA helicase		1.20	2.0E-02
M5005_Spy1448	(R[2])	hypothetical protein		1.52	4.4E-02
M5005_Spy1449		DNA primase		1.45	2.0E-02
M5005_Spy1450		phage-encoded DNA polymerase		1.45	7.7E-03
M5005_Spy1454		phage protein		1.20	1.0E-02
M5005_Spy1455		phage protein		1.60	1.5E-02
M5005_Spy1459		phage protein		2.58	1.3E-03
M5005_Spy1466		phage protein		1.29	3.2E-02
M5005_Spy1472		bis(5'-nucleosyl)-tetraphosphatase (asymmetrical)	*hit*	-1.32	2.2E-03
M5005_Spy1476		ATP/GTP hydrolase		1.16	2.2E-04
M5005_Spy1477	(R[4])	guanine-hypoxanthine permease		2.27	1.6E-05
M5005_Spy1479	*cre* (R[3])	PTS system, **Mannose**-specific IIAB; Primary Glucose PTS	*manL*	2.99	6.8E-05
M5005_Spy1480	*cre* (R[3,4])	PTS system, **Mannose**-specific IIC; Primary Glucose PTS	*manM*	3.36	1.5E-05
M5005_Spy1481	(R[3,4])	PTS system, **Mannose**-specific IID; Primary Glucose PTS	*manN*	2.78	4.1E-05
M5005_Spy1482		hypothetical cytosolic protein	*manO*	-0.97	3.1E-02
M5005_Spy1493		acyl carrier protein	*acpP*	1.39	2.0E-02
M5005_Spy1497		chaperone protein	*dnaJ*	1.27	3.3E-03
M5005_Spy1504		hypothetical membrane spanning protein		1.14	2.4E-02
M5005_Spy1508		glutamyl-tRNA(Gln) amidotransferase subunit C	*gatC*	1.04	4.3E-04
M5005_Spy1511		pyrazinamidase/nicotinamidase		1.01	3.4E-02
M5005_Spy1518		transporter		1.52	4.0E-03
M5005_Spy1519		ATP-dependent DNA helicase	*recG*	-1.14	5.2E-04
M5005_Spy1520		hypothetical protein		-4.09	7.4E-04
M5005_Spy1523		ABC transporter, ATP-binding protein	*siaF*	-1.09	6.9E-04
M5005_Spy1533		holo-[acyl-carrier protein] synthase	*alr*	1.13	8.5E-03
M5005_Spy1538	(R[2])	mannose-6-phosphate isomerase	*pmi*	-1.39	8.1E-04

M5005_Spy1539	(R[2])	fructokinase	*scrK*	1.59	4.6E-04
M5005_Spy1553		SSU ribosomal protein S18P	*rpsR*	1.93	1.2E-03
M5005_Spy1559		thioredoxin	*trx*	-1.26	2.8E-03
M5005_Spy1569	*cre*	formate acetyltransferase	*pfl*	2.57	4.0E-07
M5005_Spy1574		universal stress protein family		8.91	1.6E-10
M5005_Spy1575	*cre* (A[4])	quinolone resistance protein	*norA*	9.81	1.4E-12
M5005_Spy1581		transcriptional regulator, MerR family		1.32	8.1E-03
M5005_Spy1582		DNA polymerase III, epsilon chain	*dnaQ*	1.26	6.5E-03
M5005_Spy1598	*cre*	hypothetical protein		-1.38	2.1E-04
M5005_Spy1606		LSU ribosomal protein L28P	*rpmB*	2.04	1.7E-05
M5005_Spy1610	*cre2*	CTP synthase	*pyrG*	2.63	7.9E-06
M5005_Spy1611		DNA-directed RNA polymerase delta chain	*rpoE*	1.33	1.0E-04
M5005_Spy1627		ABC transporter permease protein	*salY*	-1.16	5.1E-04
M5005_Spy1644	(R[2])	DNA integration/recombination/invertion protein		2.93	3.1E-02
M5005_Spy1646		SSU ribosomal protein S9P	*rpsI*	1.92	3.6E-04
M5005_Spy1647		LSU ribosomal protein L13P	*rplM*	1.51	1.3E-03
M5005_Spy1661	*cre* (R[2,3,4])	transaldolase		-1.72	2.3E-03
M5005_Spy1662	(R[2,4])	putative transport protein		-1.70	1.1E-03
M5005_Spy1664	*cre* (R[2,4])	PTS system, **Mannitol**-specific IIA component		-1.17	3.2E-02
M5005_Spy1666		SSU ribosomal protein S15P		1.83	4.3E-04
M5005_Spy1667	(R[2])	hypothetical protein		1.58	1.2E-03
M5005_Spy1671		transcriptional regulator, MarR family		-1.00	8.1E-03
M5005_Spy1675		phosphatidate cytidylyltransferase	*cdsA*	-1.21	5.1E-04
M5005_Spy1691	(R[2])	exodeoxyribonuclease III		-1.63	2.1E-04
M5005_Spy1697		4-amino-4-deoxychorismate lyase		-1.09	3.0E-04
M5005_Spy1698		anthranilate synthase component II	*trpG*	-1.45	1.2E-04
M5005_Spy1699		ATPase, AAA family		-1.70	9.1E-06
M5005_Spy1710		streptococcal histidine triad protein		3.01	4.2E-08
M5005_Spy1711		laminin binding lipoprotein; AdcR- repressed	*lsp*	2.25	2.5E-05
M5005_Spy1714	(R[2])	Fn-binding surface protein; Mga-regulated	*fba*	2.51	1.9E-03
M5005_Spy1715	(R[2])	C5A peptidase; Mga-regulated	*scpA*	2.20	2.6E-03
M5005_Spy1716		transposase		-1.66	8.7E-05
M5005_Spy1717		transposase		-2.47	1.0E-03
M5005_Spy1718		secreted inhibitor of complement; Mga-regulated	*sic1.0*	2.32	2.3E-03
M5005_Spy1721	*cre*	hypothetical protein; sORF upstream of *mga*		-2.86	8.2E-03
M5005_Spy1730	(A[3])	hypothetical protein		-1.45	1.5E-04
M5005_Spy1736		hypothetical protein		-2.04	2.0E-02
M5005_Spy1737		Stand-alone transcriptional regulator Rgg, RopB	*rgg*	-1.43	1.0E-02
M5005_Spy1738	*cre* (R[3])	Secreted DNase-Streptodornase-chromosomal	*spd*	1.45	8.4E-04
M5005_Spy1741	(R[3])	glycerol dehydrogenase	*gldA*	-1.50	1.0E-04
M5005_Spy1742		transaldolase	*mipB*	-1.35	8.9E-04
M5005_Spy1743		formate acetyltransferase	*pflD*	-1.35	2.0E-05
M5005_Spy1745	(R[2,4])	PTS system, **Cellobiose**-specific IIB component	*celB*	-1.64	3.2E-02
M5005_Spy1747		sorbitol operon regulator		1.07	2.7E-02
M5005_Spy1765		cold shock protein	*csp*	1.67	7.8E-03
M5005_Spy1768	(R[4])	peroxiredoxin reductase (NAD(P)H)	*ahpC*	-1.07	6.0E-03
M5005_Spy1769	(R[4])	peroxiredoxin reductase (NAD(P)H)	*ahpF*	-1.24	5.7E-03
M5005_Spy1770	(R[2,3,4])	imidazolonepropionase	*hutI*	-5.24	1.0E-03
M5005_Spy1771	*cre* (R[2,4])	urocanate hydratase	*hutU*	-5.28	1.4E-02
M5005_Spy1773	(R[2,4])	formiminotetrahydrofolate cyclodeaminase		-7.25	3.5E-03
M5005_Spy1774	(R[2,4])	formate--tetrahydrofolate ligase	*fhs.2*	-7.37	2.1E-03
M5005_Spy1775	(R[2,4])	hypothetical cytosolic protein		-6.67	8.6 E-03
M5005_Spy1776	(R[2,4])	amino acid permease		-7.13	3.5E-03
M5005_Spy1777	(R[2,4])	histidine ammonia-lyase	*hutH*	-6.67	2.4E-03
M5005_Spy1778	(R[2])	formiminoglutamase	*hutG*	-1.82	6.1E-03
M5005_Spy1780		SSU ribosomal protein S2P	*rpsB*	1.79	3.3E-04
M5005_Spy1781		protein translation elongation factor Ts		1.72	5.7E-04
M5005_Spy1782		neutral endopeptidase	*pepO*	-1.38	2.1E-04
M5005_Spy1783		trehalose-6-phosphate hydrolase	*treC*	-1.55	3.4E-04
M5005_Spy1784		PTS system, **Trehalose**-specific IIBC component	*treB*	-0.95	3.6E-02
M5005_Spy1786		transcriptional regulator, MarR family		2.52	3.3E-04
M5005_Spy1787		glyoxalase family protein		-1.05	7.8E-03
M5005_Spy1789		anaerobic ribonucleoside-triphosphate reductase	*nrdG*	1.44	2.4E-03
M5005_Spy1790		acetyltransferase		2.23	1.4E-02
M5005_Spy1792		hypothetical protein		1.02	2.5E-03
M5005_Spy1797		hypothetical cytosolic protein		-1.38	1.2E-04
M5005_Spy1798		suppressor of clpP & X SpxA homolog, allele 2	*spxA2*	-2.61	2.3E-04
M5005_Spy1802		holliday junction DNA helicase	*ruvA*	-1.19	6.1E-04
M5005_Spy1803		multidrug resistance protein B	*lmrP*	-1.12	1.7E-03

M5005_Spy1806		hypothetical cytosolic protein		1.45	3.8E-03
M5005_Spy1810		hypothetical membrane spanning protein		1.47	6.0E-03
M5005_Spy1811		hypothetical membrane spanning protein		2.32	1.0E-02
M5005_Spy1812		hypothetical membrane spanning protein		1.18	1.1E-02
M5005_Spy1815		LSU ribosomal protein L32P	*rpmF*	2.49	1.2E-03
M5005_Spy1821		hypothetical protein		1.20	3.4E-03
M5005_Spy1823		integral membrane protein		-1.65	1.0E-05
M5005_Spy1830		transcriptional regulator, TetR family		1.12	7.0E-03
M5005_Spy1831		SSU ribosomal protein S4P	*rpsD*	1.43	2.5E-03
M5005_Spy1834		hypothetical protein		1.49	1.2E-03
M5005_Spy1840	*cre*	tRNA (5-methylaminomethyl-2-thiouridylate)-methyltransferase	*trmU*	1.32	2.4E-02
M5005_Spy1841	(A[3])	L-serine dehydratase	*sdhB*	3.26	1.1E-03
M5005_Spy1842		L-serine dehydratase	*sdhA*	3.50	5.8E-04
M5005_Spy1843	(R[2])	transglycosylase SLT domain family protein		2.76	1.2E-04
M5005_Spy1847		CDP-diacylglycerol--glycerol-3-phosphate 3-phosphatidyltransferase		-0.96	1.4E-02
M5005_Spy1849		zinc protease		-0.93	9.2E-03
M5005_Spy1851		hyaluronan synthase	*hasA*	1.30	7.9E-04
M5005_Spy1853	*cre*	UTP--glucose-1-phosphate uridylyltransferase	*hasC*	1.24	2.2E-03
M5005_Spy1862	*cre*	ABC transporter, permease protein		-1.42	4.6E-05

[a]**cre:** presence of *cre* and *cre2* sites based on Debroy et al. [1]; **(ccpA):** identifed as CcpA regulated in M1T1 transcriptome study of CcpA regulon; activated **(A)** or repressed **(R)** based on Shelburne *et al.* [2], Kinkel *et al.* [3], and Debroy *et al.* [1].

Supplemental Table S2: RNA-Seq results of the *mga* mutant in THY (WT/Δ*mga*)

Spy Number	Annotation	Gene	Log_2-FC	*p* value
M5005_Spy0012	hypoxanthine-guanine phosphoribosyltransferase		-1.73	4.0E-05
M5005_Spy0013	cell division protein	ftsH	-1.02	1.3E-03
M5005_Spy0022	phosphoribosylaminoimidazole-succinocarboxamide synthase		-1.89	1.1E-05
M5005_Spy0040	alcohol dehydrogenase	adhA	1.21	5.1E-02
M5005_Spy0041	Na+ driven multidrug efflux pump		-1.16	1.5E-04
M5005_Spy0080	bis(5'-nucleosyl)-tetraphosphatase (asymmetrical)		-1.09	4.9E-03
M5005_Spy0113	transposase		1.47	6.6E-03
M5005_Spy0115	hypothetical protein		1.21	2.2E-05
M5005_Spy0117	transcriptional regulator, LysR family		2.06	1.6E-04
M5005_Spy0118	transcriptional regulator, LysR family		1.32	2.7E-02
M5005_Spy0143	hypothetical protein		2.54	5.2E-03
M5005_Spy0156	metal-dependent hydrolase		1.16	9.1E-03
M5005_Spy0189	hypothetical protein		1.17	4.6E-02
M5005_Spy0212	N-acetylmannosamine-6-phosphate 2-epimerase		1.39	2.0E-03
M5005_Spy0213	ABC **Sialic Acid**; N-acetylneuraminate-binding protein		1.44	2.9E-03
M5005_Spy0260	putative lipase		-1.04	1.5E-03
M5005_Spy0261	GTP-binding protein		-0.98	1.8E-02
M5005_Spy0297	transposase		2.55	1.5E-02
M5005_Spy0313	riboflavin transporter		-1.20	1.4E-04
M5005_Spy0318	pyruvate formate-lyase activating enzyme	pflC	1.52	8.2E-07
M5005_Spy0319	manganese-dependent inorganic pyrophosphatase	ppaC	0.90	4.8E-05
M5005_Spy0340	L-lactate oxidase	lctO	2.47	2.6E-04
M5005_Spy0341	lactocepin; SpyCEP; IL-8 protease	spyCEP	1.69	4.9E-06
M5005_Spy0344	permease		-1.10	5.8E-04
M5005_Spy0361	phosphoglycerate transporter protein		1.01	7.8E-03
M5005_Spy0383	D-alanyl-D-alanine carboxypeptidase		-1.04	2.7E-03
M5005_Spy0432	acetyl-CoA acetyltransferase		-1.22	4.4E-04
M5005_Spy0456	plasmid stabilization system antitoxin protein		-1.13	1.1E-02
M5005_Spy0469	hypothetical protein		-1.04	4.0E-03
M5005_Spy0471	hydrolase, HAD superfamily		-1.09	3.4E-03
M5005_Spy0494	hypothetical protein		-1.19	4.3E-03
M5005_Spy0518	oligohyaluronate lyase		1.58	2.8E-03
M5005_Spy0519	PTS system, **N-acetylgalactosamine**-specific IID	agaD	1.02	3.6E-02
M5005_Spy0520	PTS system, **N-acetylgalactosamine**-specific IIC	agaS	1.89	4.9E-05
M5005_Spy0522	unsaturated glucuronyl hydrolase		1.45	8.6E-04
M5005_Spy0549	chorismate mutase		-1.15	3.8E-04
M5005_Spy0585	epuA protein	epuA	-1.20	6.4E-03
M5005_Spy0660	fructose repressor	fruR	1.06	1.8E-02
M5005_Spy0667	exotoxin type C precursor		2.07	7.1E-03
M5005_Spy0668	IgG-degrading protease	mac	1.62	6.1E-04
M5005_Spy0714	hypothetical cytosolic protein		-1.24	2.5E-04
M5005_Spy0742	hypothetical protein		-1.97	2.0E-05
M5005_Spy0743	ABC transporter substrate-binding protein		-1.59	1.8E-05
M5005_Spy0744	hypothetical protein		-1.55	2.6E-04
M5005_Spy0745	ABC transporter permease protein		-1.26	3.8E-05
M5005_Spy0746	ABC transporter ATP-binding protein		-1.47	5.9E-07
M5005_Spy0780	PTS system, **Mannose/Fructose** family IIA component	ptsA	1.50	6.8E-04
M5005_Spy0781	PTS system, **Mannose/Fructose** family IIB component	ptsB	1.30	7.2E-05
M5005_Spy0782	PTS system, **Mannose/Fructose** family IIC component	ptsC	1.03	1.4E-03
M5005_Spy0786	iron(III)-binding protein		0.89	2.0E-03
M5005_Spy0793	Xaa-His dipeptidase		-1.04	4.8E-02
M5005_Spy0797	hypothetical protein		-2.85	1.4E-02
M5005_Spy0798	IFN-response binding factor 1		-3.53	2.2E-04
M5005_Spy0824	2-amino-4-hydroxy-6-hydroxymethyldihydropteridine pyrophosphokinase	folK	-1.00	3.8E-04
M5005_Spy0834	Zn-dependent alcohol dehydrogenases and related dehydrogenases		1.20	2.6E-03

M5005_Spy0853	short chain dehydrogenase		0.96	7.1E-03
M5005_Spy0858	xanthine phosphoribosyltransferase	xpt	-2.16	7.4E-05
M5005_Spy0859	xanthine permease		-1.81	6.1E-06
M5005_Spy0914	phage transcriptional repressor		-1.32	1.4E-04
M5005_Spy0920	UDP-N-acetylmuramoylpentapeptide-lysine N(6)-alanyltransferase		-1.70	8.6E-05
M5005_Spy0921	ABC transporter ATP-binding protein		-1.54	8.4E-05
M5005_Spy0932	luciferase-like monooxygenase		0.94	1.6E-03
M5005_Spy0933	probable NADH-dependent flavin oxidoreductase		1.00	6.8E-04
M5005_Spy0934	lipoate-protein ligase A		1.07	5.1E-04
M5005_Spy0978	hypothetical protein		-1.17	6.3E-04
M5005_Spy0985	phnA protein		-1.39	6.1E-07
M5005_Spy0986	glucosamine--fructose-6-phosphate aminotransferase (isomerizing)	glmS	-1.02	5.0E-04
M5005_Spy1059	ABC **Maltodextrin** permease protein	malF	0.94	2.8E-04
M5005_Spy1062	maltodextrose utilization protein	malA	1.49	4.1E-02
M5005_Spy1063	ABC **Cyclomaltodextrin** permease protein	malD	1.54	2.7E-02
M5005_Spy1064	ABC **Cyclomaltodextrin** permease protein	malC	1.38	2.3E-02
M5005_Spy1065	alpha-amylase	amyA	1.46	1.1E-02
M5005_Spy1066	neopullulanase/cyclomaltodextrinase/maltogenic alpha-amylase	amyB	1.74	7.3E-03
M5005_Spy1067	ABC **Cyclomaltodextrin** -binding protein	malX	2.52	1.9E-05
M5005_Spy1076	Putative Bicarbonate transporter	glnH	-1.31	2.3E-03
M5005_Spy1077	Putative Bicarbonate transporter	glnQ.2	-1.04	1.3E-02
M5005_Spy1082	PTS system, **Cellobiose**-specific IIB component		1.16	3.0E-02
M5005_Spy1083	PTS system, **Cellobiose**-specific IIA component		1.00	1.1E-02
M5005_Spy1084	outer surface protein		1.02	1.8E-02
M5005_Spy1085	beta-glucosidase	bglA.2	1.46	2.3E-03
M5005_Spy1093	hypothetical protein		1.15	1.5E-02
M5005_Spy1135	oxalate/formate antiporter		-1.16	3.9E-06
M5005_Spy1148	COME operon protein 1		-1.32	2.0E-02
M5005_Spy1165	dihydroorotate dehydrogenase	pyrD	-1.19	4.5E-03
M5005_Spy1194	phage protein		-1.11	3.5E-03
M5005_Spy1235	phosphoglucomutase		1.14	9.2E-05
M5005_Spy1261	radical SAM family enzyme		-1.25	2.9E-03
M5005_Spy1274	acetyltransferase		0.90	3.7E-02
M5005_Spy1275	arginine deiminase	arcA	1.75	4.2E-03
M5005_Spy1284	cytochrome c-type biogenesis protein	ccdA	1.31	2.9E-02
M5005_Spy1299	hypothetical protein		-2.03	3.1E-03
M5005_Spy1314	hyaluronoglucosaminidase	hyl	0.85	1.0E-02
M5005_Spy1315	transcriptional regulator, GntR family		1.18	3.0E-04
M5005_Spy1316	hypothetical protein		1.35	1.9E-03
M5005_Spy1317	alpha-mannosidase		1.16	5.5E-03
M5005_Spy1340	DNA-directed RNA polymerase omega chain		-1.50	1.7E-05
M5005_Spy1347	D-beta-hydroxybutyrate dehydrogenase		-0.97	9.1E-03
M5005_Spy1376	transaldolase		1.00	5.2E-02
M5005_Spy1377	trans-acting positive regulator		1.09	5.7E-02
M5005_Spy1387	aldo/keto reductase family		1.35	2.4E-05
M5005_Spy1397	galactose-6-phosphate isomerase lacB subunit	lacB.1	1.10	5.0E-02
M5005_Spy1477	guanine-hypoxanthine permease		-1.29	2.1E-04
M5005_Spy1488	biotin carboxyl carrier protein of acetyl-CoA	accB	-1.20	1.6E-02
M5005_Spy1489	3-oxoacyl-[acyl-carrier-protein] synthase	fabF	-1.11	3.0E-02
M5005_Spy1491	malonyl-CoA-[acyl-carrier-protein] transacylase	fabD	-1.21	4.9E-03
M5005_Spy1492	enoyl-[acyl-carrier protein] reductase (NADH)	fabK	-1.13	1.8E-02
M5005_Spy1569	formate acetyltransferase	pfl	1.06	8.6E-04
M5005_Spy1572	hypothetical membrane spanning protein		-1.36	1.4E-04
M5005_Spy1587	uridine phosphorylase	udp	1.12	3.3E-05
M5005_Spy1589	transcriptional regulator, Cathelicidin resistance	crgR	-1.13	7.0E-03
M5005_Spy1618	competence-specific sigma factor	comX.2	-2.41	2.1E-02
M5005_Spy1628	ABC transporter ATP-binding protein		1.40	3.7E-03
M5005_Spy1629	lantibiotic transport ATP-binding protein	salX	1.53	4.3E-03
M5005_Spy1630	serine (threonine) dehydratase	salB	2.13	6.5E-06
M5005_Spy1631	lantibiotic salivaricin A	salA	1.81	3.4E-02

M5005_Spy1632	6-phospho-beta-galactosidase	lacG	2.06	2.7E-03
M5005_Spy1633	PTS system, **Lactose**-specific IIBC component	lacE	2.12	4.3E-03
M5005_Spy1634	PTS system, **Lactose**-specific IIA component	lacF	2.06	1.4E-02
M5005_Spy1635	tagatose-bisphosphate aldolase	lacD.2	2.11	5.4E-03
M5005_Spy1636	tagatose-6-phosphate kinase	lacC.2	2.52	1.5E-03
M5005_Spy1637	galactose-6-phosphate isomerase lacB subunit	lacB.2	2.24	4.3E-03
M5005_Spy1638	galactose-6-phosphate isomerase lacA subunit	lacA.2	1.99	2.1E-02
M5005_Spy1644	DNA integration/recombination/invertion protein		-3.39	6.7E-04
M5005_Spy1649	hypothetical membrane spanning protein		-1.00	2.9E-03
M5005_Spy1682	multiple sugar transport ATP-binding protein	msmK	1.01	1.2E-03
M5005_Spy1702	mitogenic exotoxin Z	smeZ	-1.15	1.8E-04
M5005_Spy1705	dipeptide transport system permease protein	dppB	-1.09	6.9E-03
M5005_Spy1709	hypothetical protein		-1.47	7.9E-04
M5005_Spy1714	Fn-binding surface protein; Mga-regulated	fba	3.20	4.5E-06
M5005_Spy1715	C5A peptidase; Mga-regulated	scpA	3.43	2.1E-07
M5005_Spy1717	transposase		-2.57	8.1E-06
M5005_Spy1718	secreted inhibitor of complement; Mga-regulated	sic1.0	4.63	6.2E-09
M5005_Spy1719	M protein; Mga-regulated	emm1	9.08	8.6E-16
M5005_Spy1720	Multi-virulence gene regulator Mga	mga	4.60	2.8E-15
M5005_Spy1721	hypothetical protein		-2.78	6.4E-04
M5005_Spy1733	hypothetical protein		-2.54	4.2E-03
M5005_Spy1734	streptopain SpeB inhibitor	speI	-2.94	3.1E-03
M5005_Spy1735	streptococcal pyrogenic exotoxin B	speB	-2.73	2.1E-04
M5005_Spy1738	Secreted DNase-Streptodornase-chromosomal	spd	0.98	2.2E-03
M5005_Spy1750	hypothetical protein		-1.14	2.5E-04
M5005_Spy1758	probable dipeptidase B		1.09	5.3E-04
M5005_Spy1759	transcriptional regulator, MutR family		-1.10	7.0E-03
M5005_Spy1762	10 kDa chaperonin	groES	-1.04	7.1E-03
M5005_Spy1779	transcriptional regulator, LuxR family		1.55	1.5E-03
M5005_Spy1798	suppressor of clpP & X SpxA homolog, allele 2	spxA2	-1.86	3.0E-04
M5005_Spy1809	bacteriocin	uviB	-1.13	1.5E-03
M5005_Spy1812	hypothetical membrane spanning protein		-1.01	3.0E-03
M5005_Spy1828	phage infection protein		-1.71	6.7E-07

Supplemental Table S3: RNA-Seq results of the *mga* mutant in C Media (WT/Δ*mga*)

Spy Number	Annotation	Gene	Log_2-FC	*p* value
M5005_Spy0009	hypothetical protein		1.61	4.9E-02
M5005_Spy0030	phosphoribosylaminoimidazole carboxylase catalytic subunit	purE	1.09	2.9E-02
M5005_Spy0036	protein tyrosine phosphatase		-0.93	4.0E-03
M5005_Spy0091	comG operon protein 6	comYD	1.45	1.4E-02
M5005_Spy0106	transcriptional regulator; Regulator of protein F	rofA	-1.09	1.8E-04
M5005_Spy0113	transposase		1.32	1.8E-02
M5005_Spy0129	V-type ATP synthase subunit C	ntpC	-1.78	6.7E-03
M5005_Spy0130	V-type sodium ATP synthase subunit F	ntpF	-1.44	8.8E-03
M5005_Spy0131	V-type sodium ATP synthase subunit A	ntpA	-1.30	1.4E-02
M5005_Spy0132	V-type sodium ATP synthase subunit B	ntpB	-1.50	1.2E-02
M5005_Spy0133	V-type sodium ATP synthase subunit D	ntpD	-1.57	8.5E-03
M5005_Spy0136	adenylosuccinate synthetase	purA	-1.11	2.9E-05
M5005_Spy0139	NAD glycohydrolase	nga/spn	1.04	4.6E-02
M5005_Spy0140	NAD glycohydrolase inhibitor	ifs	1.14	4.9E-02
M5005_Spy0141	streptolysin O	slo	1.12	2.9E-02
M5005_Spy0143	hypothetical protein		3.35	2.1E-03
M5005_Spy0157	glycine betaine transport ATP-binding protein	opuAA	1.23	4.8E-06
M5005_Spy0167	transposase		1.58	2.9E-02
M5005_Spy0183	hypothetical membrane associated protein		0.91	5.4E-03
M5005_Spy0202	hypothetical protein		-1.34	6.9E-05
M5005_Spy0247	D-alanyl-D-alanine carboxypeptidase		0.99	1.3E-04
M5005_Spy0257	transposase		-1.55	2.2E-02
M5005_Spy0279	hypothetical protein	lemA	-0.91	6.3E-04
M5005_Spy0296	acylphosphatase		-1.04	2.9E-04
M5005_Spy0385	67 kDa Myosin-crossreactive antigen		-1.32	6.1E-05
M5005_Spy0403	hypothetical protein		0.92	4.6E-02
M5005_Spy0422	NAD(P)H-dependent quinone reductase		-1.11	4.8E-05
M5005_Spy0423	Xaa-Pro dipeptidase	pepQ	-1.23	4.7E-05
M5005_Spy0477	hypothetical membrane spanning protein	scfB	-0.97	2.8E-04
M5005_Spy0478	hypothetical membrane spanning protein	scfA	-1.08	6.0E-04
M5005_Spy0486	hypothetical protein		-1.08	2.6E-04
M5005_Spy0522	unsaturated glucuronyl hydrolase		0.98	5.0E-03
M5005_Spy0527	4-hydroxy-2-oxoglutarate aldolase/	kgdA	1.03	5.4E-04
M5005_Spy0555	hypothetical cytosolic protein		-1.09	2.7E-04
M5005_Spy0560	transcriptional regulator		-0.94	1.4E-02
M5005_Spy0562	streptolysin S precursor	sagA	1.33	7.4E-04
M5005_Spy0564	streptolysin S biosynthesis protein	sagC	0.99	6.8E-04
M5005_Spy0565	streptolysin S biosynthesis protein	sagD	1.09	1.5E-04
M5005_Spy0567	streptolysin S biosynthesis protein	sagF	1.46	1.1E-04
M5005_Spy0568	streptolysin S export ATP-binding protein	sagG	1.30	2.5E-05
M5005_Spy0569	streptolysin S export transmembrane protein	sagH	1.09	3.4E-04
M5005_Spy0570	streptolysin S export transmembrane protein	sagI	1.42	1.3E-04
M5005_Spy0571	endonuclease/exonuclease/phosphatase family protein		1.21	1.1E-06
M5005_Spy0598	large-conductance mechanosensitive channel	mscL	-0.88	2.6E-02
M5005_Spy0615	pore forming protein	ebsA	-1.03	9.6E-04
M5005_Spy0616	ferredoxin		-2.15	1.1E-03
M5005_Spy0641	aspartate carbamoyltransferase	pyrB	0.90	5.7E-03
M5005_Spy0651	Heme-utilization cell surface protein	hupY	2.28	4.6E-09
M5005_Spy0652	Heme-oxygenase cytosolic protein	hupZ	2.16	5.2E-08
M5005_Spy0668	IgG-degrading protease	mac	1.37	1.2E-02
M5005_Spy0679	GTP pyrophosphokinase		-1.01	3.0E-04
M5005_Spy0703	orotidine 5'-phosphate decarboxylase	pyrF	1.15	9.2E-03
M5005_Spy0708	uracil-DNA glycosylase	ung	-0.86	4.1E-02
M5005_Spy0715	SSU ribosomal protein S1P		1.28	9.4E-04
M5005_Spy0720	putative exfoliative toxin		0.86	1.1E-02

M5005_Spy0753	dihydrolipoamide acetyltransferase pyruvate dehydrogenase complex	acoC	-0.85	8.2E-04
M5005_Spy0754	hypothetical protein		-1.50	5.8E-02
M5005_Spy0755	dihydrolipoamide dehydrogenase	acoL	-1.16	2.9E-05
M5005_Spy0794	tRNA (5-carboxymethylaminomethyl-2-thiouridylate) synthase	thdF	1.10	2.7E-04
M5005_Spy0820	tetrahydropholate synth. /dihydrofolate synthase	folC.1	-0.98	2.0E-05
M5005_Spy0821	tetrahydropholate synth., GTP cyclohydrolase I	folE	-0.95	6.7E-05
M5005_Spy0822	tetrahydropholate synth., dihydropteroate synthase	folP	-0.92	5.7E-03
M5005_Spy0824	tetrahydropholate synth., pyrophosphokinase	folK	-0.87	1.4E-3
M5005_Spy0879	hypothetical protein		-1.52	7.9E-05
M5005_Spy0880	conserved membrane protein		-2.45	2.5E-05
M5005_Spy0881	hypothetical cytosolic protein		-1.93	5.2E-05
M5005_Spy0890	D-lactate dehydrogenase	ddh	1.38	5.2E-03
M5005_Spy0898	2-(5''-triphosphoribosyl)-3'-dephosphocoenzyme-A synthase		1.05	3.0E-02
M5005_Spy1005	phage protein		-1.47	2.1E-02
M5005_Spy1035	phage protein		1.44	6.0E-02
M5005_Spy1076	Putative Bicarbonate transporter	glnH	-0.85	3.2E-02
M5005_Spy1077	Putative Bicarbonate transporter	glnQ.2	-1.04	1.0E-02
M5005_Spy1093	hypothetical protein		-1.16	2.0E-04
M5005_Spy1145	superoxide dismutase	sodA	-0.93	5.7E-03
M5005_Spy1169	phage-encoded streptodornase; secreted DNase	spd3	2.35	1.0E-06
M5005_Spy1170	hypothetical membrane associated protein		2.39	6.1E-07
M5005_Spy1199	phage protein		-1.87	9.6E-03
M5005_Spy1211	phage protein		1.19	5.0E-02
M5005_Spy1213	phage protein		1.28	7.5E-03
M5005_Spy1227	hypothetical protein		1.06	6.2E-05
M5005_Spy1228	DNA repair protein	recN	0.99	6.7E-03
M5005_Spy1229	arginine repressor	argR1	0.99	3.2E-02
M5005_Spy1230	hemolysin		0.92	3.2E-02
M5005_Spy1237	arginine transport ATP-binding protein	artP	1.36	7.8E-04
M5005_Spy1238	arginine transport system permease protein	artQ	1.40	2.1E-04
M5005_Spy1239	hypothetical cytosolic protein		1.14	1.5E-02
M5005_Spy1285	CRISPR2-associated protein Cas2	cas2	1.11	4.9E-04
M5005_Spy1287	CRISPR2-associated protein Cas4	cas4	1.08	2.6E-04
M5005_Spy1289	CRISPR2-associated protein Cds1	cds1	1.06	4.0E-07
M5005_Spy1297	phospho-2-dehydro-3-deoxyheptonate aldolase		1.18	3.0E-03
M5005_Spy1320	regulatory protein	recX	-1.55	1.4E-05
M5005_Spy1323	transposase		0.96	9.6E-03
M5005_Spy1325	ribosome-associated factor Y		-1.16	5.8E-04
M5005_Spy1369	hypothetical protein		1.18	2.4E-02
M5005_Spy1372	ABC transporter permease protein	proB	1.11	2.2E-04
M5005_Spy1382	hypothetical membrane associated protein		-1.40	1.4E-03
M5005_Spy1400	PTS system, Galactose-specific IIB component		-2.47	2.4E-03
M5005_Spy1401	PTS system, Galactose-specific IIA component		-2.66	2.9E-04
M5005_Spy1407	streptococcal secreted esterase Sse	sse	1.81	6.5E-04
M5005_Spy1415	phage-encoded streptodornase; Secreted DNase	sdaD2	2.22	8.7E-06
M5005_Spy1440	terminase large subunit		1.23	1.2E-02
M5005_Spy1441	phage terminase small subunit		1.12	3.4E-02
M5005_Spy1514	universal stress protein family		-1.22	1.2E-03
M5005_Spy1525	conserved hypothetical protein	siaD	1.09	2.2E-03
M5005_Spy1526	ABC transporter, ATP-binding protein, putative	siaC	1.33	7.5E-07
M5005_Spy1527	ABC transporter, ATP-binding protein, putative	siaB	1.39	1.2E-03
M5005_Spy1528	iron compound ABC transporter, ATP-binding protein	siaA	1.44	9.5E-04
M5005_Spy1529	iron compound ABC transporter, permease protein	shp	1.22	6.6E-05
M5005_Spy1530	iron compound ABC transporter, substrate-binding protein	shr	1.27	4.4E-07
M5005_Spy1559	thioredoxin	trx	-0.96	9.7E-04
M5005_Spy1590	SSU ribosomal protein S14P	rpsN2	-1.22	7.5E-03
M5005_Spy1622	type I restriction-modification system specificity subunit	hsdS	1.20	9.4E-07
M5005_Spy1714	Fn-binding surface protein; Mga-regulated	fba	4.79	8.7E-08
M5005_Spy1715	C5A peptidase precursor; Mga-regulated	scpA	5.04	2.9E-10

M5005_Spy1716	transposase		-1.07	1.3E-04
M5005_Spy1718	secreted inhibitor of complement; Mga-regulated	sic1.0	5.53	2.1E-09
M5005_Spy1719	M protein; Mga-regulated	emm1	10.06	8.1E-17
M5005_Spy1720	Multi-virulence gene regulator Mga	mga	5.84	4.2E-17
M5005_Spy1721	hypothetical protein		-5.00	2.4E-08
M5005_Spy1727	ABC transporter ATP-binding protein		0.99	1.0E-02
M5005_Spy1731	gene regulated by Mga; Mga-regulated	grm	3.85	7.7E-05
M5005_Spy1733	hypothetical protein		1.45	5.2E-02
M5005_Spy1736	hypothetical protein		-1.31	3.0E-02
M5005_Spy1738	Secreted DNase-Streptodornase-chromosomal	spd	1.03	3.2E-04
M5005_Spy1739	hypothetical protein		-3.02	3.2E-05
M5005_Spy1754	translation initiation inhibitor		-0.98	4.6E-02
M5005_Spy1768	peroxiredoxin reductase (NAD(P)H)	ahpC	-0.92	1.1E-03
M5005_Spy1774	formate--tetrahydrofolate ligase	fhs.2	1.84	2.5E-02
M5005_Spy1775	hypothetical cytosolic protein		1.59	5.0E-02
M5005_Spy1776	amino acid permease		1.83	2.8E-02
M5005_Spy1777	histidine ammonia-lyase	hutH	2.16	9.1E-03
M5005_Spy1778	formiminoglutamase	hutG	1.01	9.6E-03
M5005_Spy1798	suppressor of clpP & X SpxA homolog, allele 2	spxA2	-1.07	1.7E-02
M5005_Spy1823	integral membrane protein		-0.88	3.5E-04
M5005_Spy1843	transglycosylase SLT domain family protein		1.33	4.5E-03

Supplemental Table S4: PCR primers used in this study

Target	Primer Name	Sequence (5'-3')† ^	Reference
qPCR			
gyrA	gyrA M1 RT-L	CGACTTGTCTGAACGCCAAAGT	[4]
	gyrA M1 RT-R	ATCACGTTCCAAACCAGTCAAAC	[4]
emm	emm1 RT-L	ACTCCAGCTGTTGCCATAACAG	[5]
	emm1 RT-R	GAGACAGTTACCATCAACAGGTGAA	[5]
sclA	sclA M1 RT-L	TGCTGACAAAGAAGCTAACCAAAC	[5]
	sclA M1 RT-L	CGTCTGTGGTTGTTGGCTACAG	[5]
fruA	fruA M1 RT-L	ACCGGGCTTAGTAGCTGGT	[6]
	fruA M1 RT-R	ACTTCTCCTCCTGCTGCAA	[6]
sdaD2	sdaD2 M1T1 RT-L	AGCAAACCAGAGCCAAGTG	This study
	sdaD2 M1T1 RT-R	CACATTTTTGGGGCTATGC	This study
speB	speB M1 RT-L	GGTAAAGTAGGCGGACATGCC	[7]
	speB M1 RT-R	CACCCCAACCCCAGTTAACA	[7]
salB	salB M1T1 RT-L	TGGTGTTTTAGCAGCATTTT	This study
	salB M1T1 RT-R	AATCTCCGCCTGAAACAAT	This study
mac	mac M1T1 RT-L	ACGGCTACCGCCTTAGTCT	This study
	mac M1T1 RT-R	ACGGCGTCAAAAATACCAC	This study
opuAA	opuAA M1 RT-L	TGATTTGCAAGACAGCATGA	[7]
	opuAA M1 RT-R	CATCAAAGCAATCCGATCAC	[7]
ska	ska M1T1 RT-L	TCGAAACGGCAAGGTCTAC	This study
	ska M1T1 RT-R	GCGCACATGTCCCTTTAAC	This study
sagC	sagC M1 RT-L	TGCTGATGCTTGAGGATGAC	[3]
	sagC M1 RT-R	GCGCCATAAAACGGAAATTA	[3]
glnH	glnH M1 RT-L	TGAGGGCATTTCTACTGGTG	This study
	glnH M1 RT-R	GCAGCTAAGCCATTGTTGAA	This study
slo	slo M1 RT-L	TTGTTGAGGATAATGTAAGAATGTTTAG	[3]
	slo M1 RT-R	TCCTGGCTTGCAACTGATTG	[3]
PCR			
mga	Mga InIn F	ccc**GGATCC**CAGTGGAGAGAACTAAAATT	This study
	Mga InIn R	ccc**GGATCC**TAATTCTTCCAAAGAGTTGA	This study
M13	1201	AACAGCTATGACCATGATTACG	Genewiz
	1211	GTTGTAAAACGACGGCCAGT	Genewiz
aad9	SPR1	*ccc***CTCGAG**AATAAAGCCCGCTTCGGCG	[8]
	SPR2	*ccc***ACTAGT**AAGGACTTAAAAGAGCGTATTG	[8]

† Bold type denotes restriction site

^ Lowercase letters denote nucleotides not complementary to target DNA

Supplemental References

1 DebRoy, S. *et al.* A Multi-Serotype Approach Clarifies the Catabolite Control Protein A Regulon in the Major Human Pathogen Group A Streptococcus. *Sci. Rep.* **6**, 32442, doi:10.1038/srep32442 (2016).

2 Shelburne, S. A., 3rd *et al.* A direct link between carbohydrate utilization and virulence in the major human pathogen Group A Streptococcus. *Proc. Natl. Acad. Sci. USA* **105**, 1698-1703 (2008).

3 Kinkel, T. L. & McIver, K. S. CcpA-mediated repression of streptolysin S expression and virulence in the Group A Streptococcus. *Infect. Immun.* **76**, 3451-3463 (2008).

4 Ribardo, D. A., Lambert, T. J. & McIver, K. S. Role of *Streptococcus pyogenes* two-component response regulators in the temporal control of Mga and the Mga-regulated virulence gene *emm*. *Infect. Immun.* **72**, 3668-3673 (2004).

5 Beres, S. B. *et al.* Molecular genetic anatomy of inter- and intraserotype variation in the human bacterial pathogen Group A Streptococcus. *Proc. Natl. Acad. Sci. USA* **103**, 7059-7064 (2006).

6 Valdes, K. M. *et al.* The *fruRBA* operon is necessary for Group A Streptococcal growth in fructose and for resistance to neutrophil killing during growth in whole human blood. *Infect. Immun.*, doi:10.1128/IAI.01296-15 (2016).

7 Leday, T. V. *et al.* TrxR, a new CovR-repressed response regulator that activates the Mga virulence regulon in Group A Streptococcus. *Infect. Immun.* **76**, 4659-4668 (2008).

8 Le Breton, Y. *et al.* Genome-wide identification of genes required for fitness of Group A Streptococcus in human blood. *Infect. Immun.* **81**, 862-875 (2013).
